# Experimental and Theoretical Studies of a Spirostannole and Formation of a Pentaorganostannate †

**DOI:** 10.3390/molecules25214993

**Published:** 2020-10-28

**Authors:** Isabel-Maria Ramirez y Medina, Markus Rohdenburg, Waldemar Kipke, Enno Lork, Anne Staubitz

**Affiliations:** 1 MAPEX Center for Materials and Processes, University of Bremen, Bibliothek Str. 1, 28359 Bremen, Germany; ramirez-y-medina@uni-bremen.de (I.-M.R.y.M.); m.rohdenburg@uni-bremen.de (M.R.); kipke@uni-bremen.de (W.K.); elo@uni-bremen.de (E.L.); 2 Institute for Organic and Analytical Chemistry, University of Bremen, Leobener Str. 7, 28359 Bremen, Germany; 3 Institute for Applied and Physical Chemistry, University of Bremen, Leobener Str. 5, 28359 Bremen, Germany; 4 Institute for Inorganic Chemistry and Crystallography, University of Bremen, Leobener Str. 7, 28359 Bremen, Germany

**Keywords:** organometallics, stannoles, spiro compounds, density functional calculations, main group chemistry

## Abstract

A new spirostannole, 1,1′,3,3′-tetrakis(5-methylthiophen-2-yl)-4,4′,5,5′,6,6′,7,7′-octahydro-2,2′-spirobi[benzo[*c*]stannole] (**4**), is synthesised and the molecular structure is compared with the optimised geometry from DFT calculations. The highest occupied molecular orbital (HOMO) and lowest unoccupied molecular orbital (LUMO) are twice degenerated and show a small HOMO–LUMO energy gap of 3.2 eV. In addition, cyclic voltammetry measurements are conducted and three redox processes are observed. Absorption and emission spectra show maxima at λ_abs,max_ 436 nm and λ_em,max_ 533 nm, respectively. Spirostannole **4** is a strongly absorbing material, but an extremely weak emitter in solution at 295.15 K. However, when the solution is cooled from 280 to 80 K, the emission becomes visible. The reaction of spirostannole **4** with methyllithium is monitored by NMR spectroscopy at 238.15 K. The ^119^Sn{^1^H} NMR signal shifts from −36.0 (**4**) to −211.0 ppm, which is indicative of the formation of the lithium pentaorganostannate **5**. The complex is thermally instable at 295.15 K, but insights into the molecular structure and electronic behaviour are obtained by DFT and TD-DFT calculations.

## 1. Introduction

In general, stannoles represent cyclic organotin compounds and belong to the group 14 metalloles. Their structure is analogous to the cyclopentadienes but with a stannylene moiety instead of the methylene moiety. Stannoles, both classical and annulated, have received increasing interest during the last 20 to 30 years because of their unusual optoelectronic properties, their small highest occupied molecular orbital (HOMO)—lowest unoccupied molecular orbital (LUMO) energy gap and their potential applications in devices [1,2,3,4].

So far, only their Si and Ge congeners, the siloles and germoles, have been used as materials in electronic devices and have been extensively studied [5,6]. Stannoles, on the other hand, have been investigated less often. However, as different high-yielding synthetic methods have emerged, their optical, electronic, and structural properties and use as building blocks in materials have become a focus of research [2,3,4,7,8,9,10,11,12,13,14,15,16,17]. Aside from their innate properties, their reactivity is also of interest. 

The tin–lithium exchange reaction is an efficient and very mild method to generate organolithium compounds that cannot be synthesised by traditional halogen–lithium exchange reactions or only proceed with little conversion [18,19,20,21,22,23,24]. Because of this, it is of interest to compare the often-used linear organotin reagents with the cyclic stannoles concerning their reactivity with MeLi. The intermediates in tin–lithium exchange reactions are lithium pentaorganostannates with a tin atom bearing five carbon substituents. In almost all reported cases, these are only stable at lower temperatures or have to be stabilised by additives [20,25,26]. So far, just one structure is known that is stable at room temperature and that could be isolated by crystallisation with 1,2-dimethoxyethane (DME) and analysed by X-ray crystallography [20,25,27,28]. 

In this report, the synthesis and experimental and theoretical characterisation of a new spirostannole, 1,1′,3,3′-tetrakis(5-methylthiophen-2-yl)-4,4′,5,5′,6,6′,7,7′-octahydro-2,2′-spirobi[benzo[*c*]stannole] (**4**), are presented. In addition, the reaction of stannole **4** with methyllithium is demonstrated, leading to a pentavalent “ate” complex **5**. Calculations regarding the optimised geometry, orbital energies and absorption were also conducted for the lithium pentaorganostannate **5** to furnish insights into its interesting and unique structure.

## 2. Results and Discussion

**Scheme 1:**
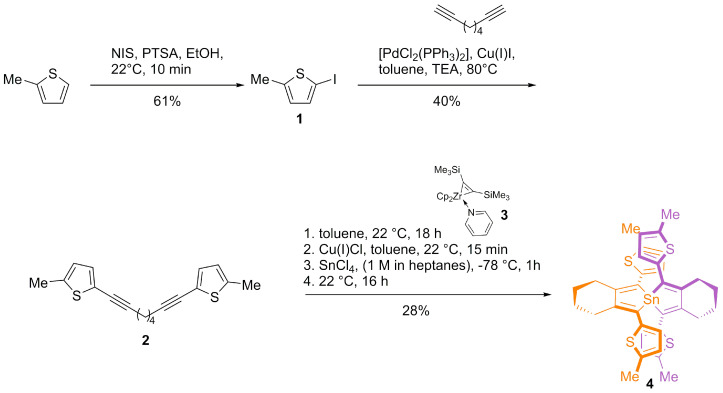
Synthesis of spirostannole 1,1′,3,3′-tetrakis(5-methylthiophen-2-yl)-4,4′,5,5′,6,6′,7,7′-octahydro-2,2′-spirobi[benzo[*c*]stannole] (**4**) and its precursor molecules. PTSA = *p*-toluenesulfonic acid, TEA = triethylamine.

The precursor molecule 1,8-bis(5-methyl-thiophen-2-yl)octa-1,7-diyne (**2**) was synthesised by an electrophilic aromatic substitution of 2-methylthiophene with *N*-iodosuccinimide (NIS) in the first step followed by a Sonogashira cross coupling reaction of 2-iodo-5-methylthiophene (**1**) with 1,7-octadiyne in the second step [29]. Cyclisation of 1,8-bis(5-methylthiophen-2-yl)octa-1,7-diyne (**2**) with Rosenthal´s zirconocene (**3**) produced a zirconacyclopentadiene as an intermediate, which was treated with copper(I) chloride and tin(IV) chloride to furnish the spirostannole **4** with a yield of 28% (Scheme 1); for experimental procedures, see the Electronic Supporting Information (ESI) [30,31]. 

**Figure 1:**
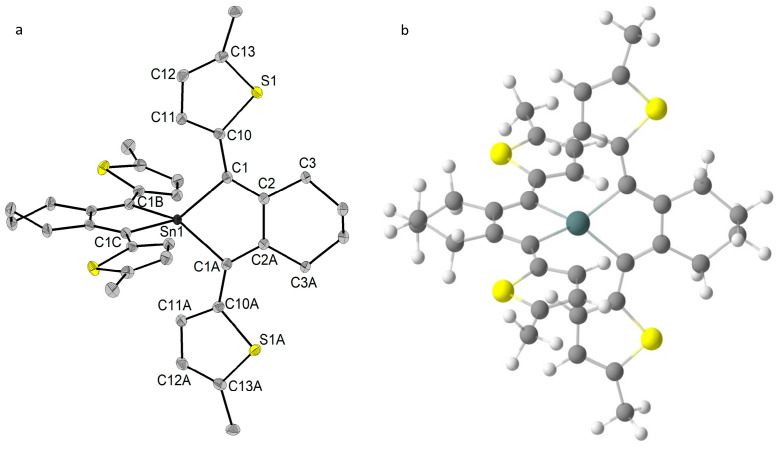
(**a**) Molecular structure of spirostannole **4**, showing 50% probability ellipsoids and the crystallographic numbering scheme. All hydrogen atoms were omitted for clarity. (**b**) Optimised geometry of spirostannole **4** based on DFT calculations.

Spirostannole **4** could be crystallised from toluene and gave orange-yellow single crystals. An X-ray crystal analysis confirms the molecular structure and identity of compound **4**. The Sn atom is incorporated into two planar stannole rings (Sn1-C1-C2-C2A 1.8(3)°), which are twisted against each other with a torsion angle of C2-C1-Sn1-C1B −125.38(3)°. The stannole-thiophenyl systems are almost planar (Sn1-C1-C10-C11 13.7(3)°) and the annulated six-membered rings exhibit a puckered conformation (Figure 1). Crystal data and bond lengths and bond angles are provided in the ESI in Tables S3–S5.

**Figure 2:**
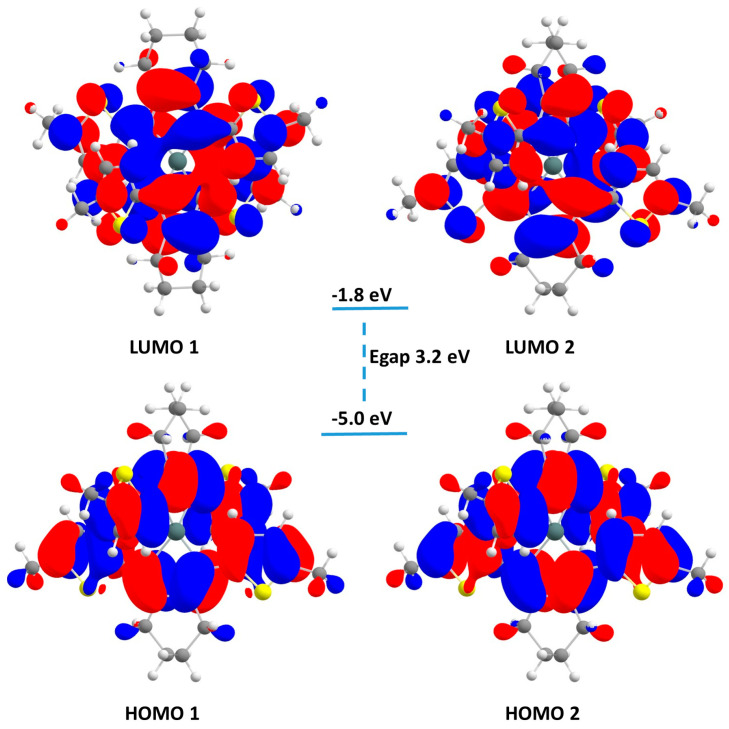
FMOs and HOMO-LUMO energy gap of spirostannole **4** based on DFT calculations.

The geometry optimisation by DFT calculations (PBE1PBE-GD3BJ/6-311++G(2d,2p) with an SDD basis for Sn) agrees with the experimental results [32,33,34,35,36,37,38,39,40,41]. This level of theory was chosen because it showed sufficient agreement with computational data on the electronic structure and transitions in another study of stannoles [17]. An investigation into the frontier molecular orbitals (FMOs) revealed that both the HOMO and LUMO are twice degenerated due to the symmetry of the molecule. Both the HOMOs and LUMOs are delocalised over the whole backbone, but the annulated cyclohexane ring is not involved. In comparison to other stannoles, the Sn atom is not involved in the LUMOs [1]. So far, this phenomenon has only been observed twice for stannoles (see our previous reports) [15,17]. The HOMO-LUMO energy gap was calculated as 3.2 eV (Figure 2) and is still in the same range as that for other stannoles [15,17].

**Figure 3:**
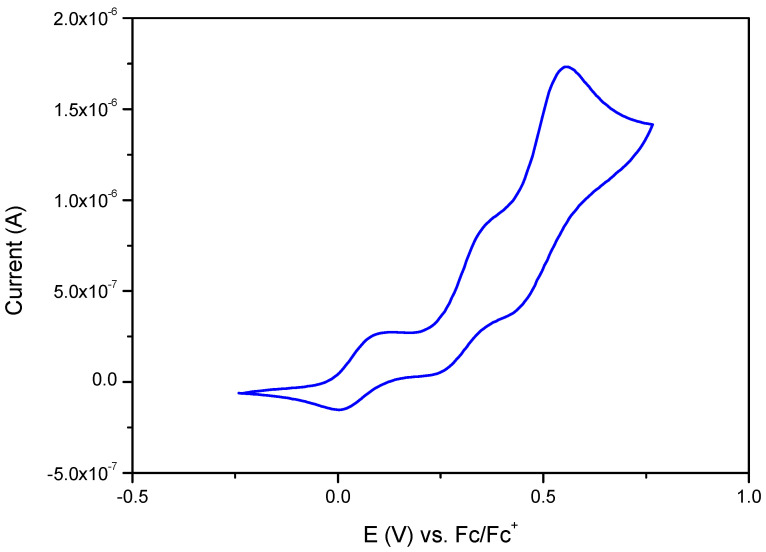
Quasi-reversible redox processes of stannole **4** at 298 K. The conducting salt was 0.1 M Bu_4_NPF_6_ in dichloromethane and the scan rate was 0.2 V/s. The spectrum is referenced against F/Fc^+^.

To gain more insight into the electrochemical behaviour of spirostannole **4**, we additionally conducted cyclic voltammetry measurements (Figure 3). The anodic scan was in the range of −240 to +765 mV with a scan rate of 0.2 V/s. The spectrum seems to exhibit three quasi-reversible redox processes with |∆E^1^_p_| = 90.1 mV, |∆E^2^_p_| = 115.6 mV and |∆E^3^_p_| = 116.4 mV, respectively (all values were referenced against Fc/Fc^+^, Table 1). Although the cyclic voltammogram does not seem to be completely reversible, the intensities of E_pa_ and E_pc_ were still stable after multiple scans when an equilibrium was reached. The compound did not decompose. The reason for the high differences between E_pa_ and E_pc_ of each redox-couple might be the existence of the double degenerated FMO´s. Furthermore, from the first redox wave to the second and then the third, the amount of current increased strongly (Figure 3).

**Figure 4:**
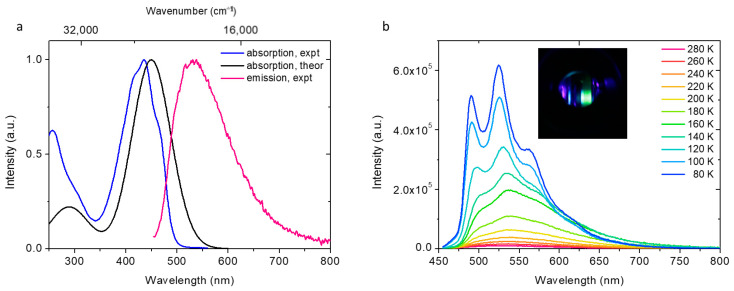
(**a**) Experimental absorption/emission spectra in chloroform and the calculated absorption spectrum. (**b**) Emission spectra at different temperatures in 2-methyl-tetrahydrofuran and a picture of the irradiated solution of spirostannole **4** (green luminescence) in 2-methyltetrahydrofuran at 80 K.

The optical properties of spirostannole **4** were supported by UV–Vis and fluorescence measurements. Furthermore, the experimental absorption data were compared with data from TD-DFT calculations. The experimental absorption and emission maxima were at λ_abs,max_ 436 nm and λ_em,max_ 533 nm in chloroform at 295.15 K. A second absorption maximum was in the higher energy region of the spectrum at 257 nm. The stoke shift is therefore 4174 cm^−1^ (Figure 4a).

The TD-DFT analysis produced a main absorption maximum at 450 nm, which is shifted about 14 nm compared with the experimentally measured absorption. This absorption maximum could be attributed to the HOMO 2→LUMO 1, HOMO 1→LUMO 2, HOMO 2→LUMO 2, and HOMO 1→LUMO 1 transitions including the π-skeleton of the stannole ring. The lower absorption maximum was located at 289 nm (Figure 4a).

Spirostannole **4** is an extremely weak emitter with Φ_FL_ < 0.1% but strongly absorbing with ε = 31,235 L∙mol^−1^∙cm^−1^. However, the photoluminescence in 2-methyltetrahydrofuran at 80 K was stronger by a factor of 59 compared to 280 K and became visible with green luminescence (Figure 4b). The half-life at 80 K was τ = 1.205 ns (21.59%), 3.909 ns (78.41%) but we did not observe phosphorescence. These results are similar to our previous investigations reported recently [17].

**Scheme 2:**
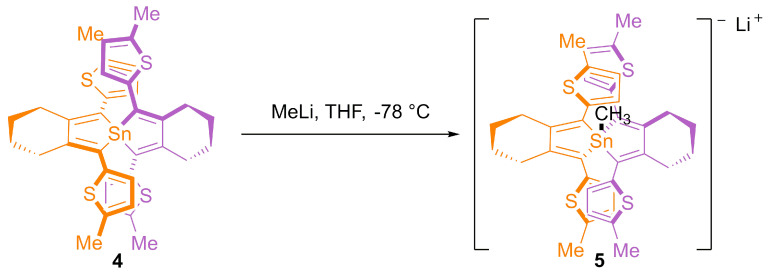
NMR experiment: reaction of spirostannole **4** with MeLi under inert conditions.

In addition, we studied the reaction of spirostannole **4** with methyllithium [27,28]. Under inert conditions, a solution of methyllithium was added to a solution of spirostannole **4** in tetrahydrofuran (THF) at 195.15 K in an inert NMR tube (Scheme 2). The colour changed immediately from orange-yellow to red.

**Figure 5:**
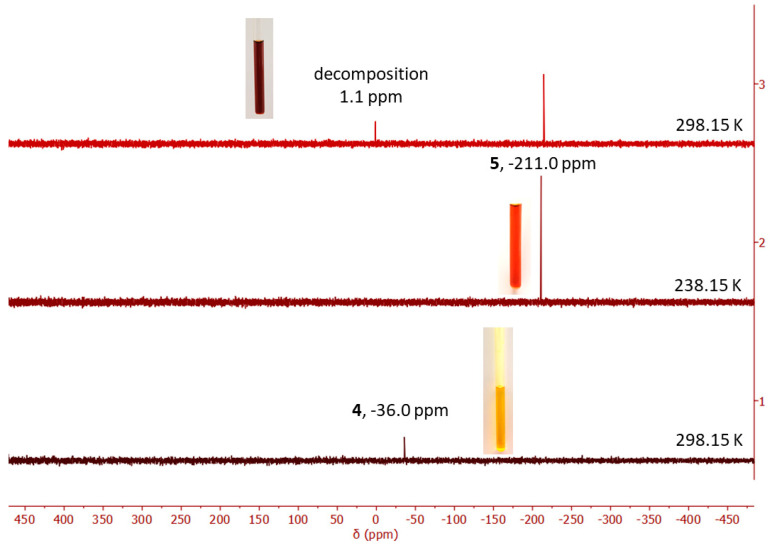
^119^Sn{^1^H} NMRs of stannole **4** stannolate **5**, and the decomposition.

Monitoring of the ^119^Sn{^1^H} and ^7^Li NMR signals at 238.15 K (this was the minimum reachable temperature of the 600 MHz NMR device) proved the formation of the pentaorganostannate complex **5**: the ^119^Sn{^1^H} signal shifted from −36.0 ppm (spirostannole **4**) to −211.0 ppm (complex **5**). This is in agreement with the findings of Saito and co-workers regarding their lithium pentaorganostannate (Figure 5) [27,28]. The ^7^Li NMR signal shifted from 1.8 ppm (for MeLi) to −0.4 ppm. The ^1^H NMR spectrum showed a broad Sn-CH_3_ signal at −0.11 ppm.

Warming the reaction mixture to 298.15 K led to a decomposition of both the complex and spirostannole **4**, the ^119^Sn{^1^H} NMR signal shifted to 1.1 ppm (Figure 5).

After ~1 min at 298.15 K, the solution turned dark brown and was turbid brownish-yellow with a slight precipitate after several hours. This means that the pentaorganostannate **5** is only stable at lower temperatures. A possible reason could be the structural difference between classical stannoles and annulated ring systems such as stannafluorenes, which might be associated with differences in stability [27,28]. 

**Figure 6:**
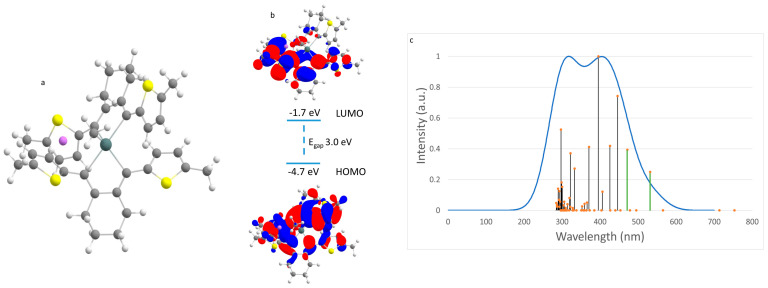
(**a**) Optimised geometry of pentaorganostannate **5**; (**b**) FMOs of pentaorganostannate **5** and energies; (**c**) Calculated TD-DFT absorption spectrum of pentaorganostannate **5**. Direct HOMO→LUMO transitions are highlighted in green.

To obtain insight into the molecular structure and opto-electronics, we conducted DFT and TD-DFT calculations (PBE1PBE-GD3BJ/6-311++G(2d,2p) with an SDD basis for Sn) (Figure 6a–c) [32,33,34,35,36,37,38,39,40,41]. Stannolate **5** exhibits a (distorted) trigonal bipyramidal geometry similar to the pentaorganostannate of Saito and co-workers and other pentacoordinated stannates, but less distorted with more ideal bond angles around the tin atom [8,25,27,28]. The C(ax)–Sn–C(ax) and C(eq)–Sn–C(eq) bond angles are 179.4° and 121.0°, respectively. The bond lengths of C(ax_1,2_)—Sn, C(eq_1,2_)—Sn and C(Me)—Sn are 2.321/2.242, 2.193/ 2.216 and 2.270 Å, respectively. The thiophenyl–stannole–thiophenyl system is no longer planar with the thiophenyl rings twisted against the central stannole ring with different torsion angles in the range of 126.8° to −164.8°. Although both stannole rings were twisted against each other, but on the same plane in **4**, the stannole rings are bent out of the plane because of the extra methyl group on the Sn in **5** (Figure 1 and Figure 6a).

As expected, the double degeneration of the frontier molecular orbitals (FMOs) of stannole **4** is cancelled by the symmetry break in pentaorganostannate **5**. Though the FMOs are located over both stannole rings in spirostannole **4**, they are not in compound **5**. The HOMO is mainly located on one stannole ring and the LUMO is mainly localised on the other ring. The energy gap of 3.0 eV is slightly smaller than that of stannole **4**. The calculated absorption spectrum of **5** with all transitions is illustrated in Figure 6c. Two maxima could be observed, one at λ_abs,max_ 317 nm and the other one at λ_abs,max_ 405 nm. However, there are many transitions; the most significant are listed in Table S6 in the ESI and all the involved orbitals are depicted (see ESI).

## 3. Materials and Methods

Reactions under inert conditions were carried out using standard Schlenk techniques under a dry, inert N_2_ or Ar atmosphere or in a N_2_-filled glovebox from Inert (Inert technology, Amesbury, MA, USA). All anhydrous solvents were taken from the solvent purification system (SPS), degassed by four freeze-pump-thaw cycles and stored under a N_2_ atmosphere. All chemicals were commercially available and were used without further purification unless noted otherwise. ^1^H, ^13^C{^1^H}, ^7^Li and ^119^Sn{^1^H} NMR spectra were recorded on a Bruker Avance Neo 600 or Bruker DRX 500 (Bruker, Billerica, MA, USA) at 25 °C unless noted otherwise. Electron impact (EI) ionisation mass spectra were obtained on the double-focusing mass spectrometer MAT 95XL from Finnigan MAT (Thermo Fisher Scientific, Waltham, MA, USA). IR spectra were recorded on a Nicolet Thermo IS10 SCIENTIFIC spectrometer (Thermo Fisher Scientific, Waltham, MA, USA) with a diamond ATR unit. All melting points were measured with a Büchi Melting Point M-560 apparatus (Büchi Labortechnik GmbH, Essen, Germany) and are uncorrected.

UV-Vis spectra were recorded at a resolution of 0.1 nm on a UV-2700 spectrometer from Shimadzu (Shimadzu, Kyoto, Japan).

Emission spectra were recorded on an Edinburgh Instruments FLS 1000 photoluminescence spectrometer (Edinburgh Instruments Ltd, Livingston, Scotland). Absolute quantum yields (QYs) were measured with an Edinburgh Instruments integrating sphere (Edinburgh Instruments Ltd, Livingston, Scotland). All emission spectra are corrected spectra. Cyclic voltammetry measurements were recorded using the potentiostat Autolab PGSTAT101 Metrohm (Methrom AG, Herisau, Switzerland). The working electrode and the counter electrode were platinum. The material of the reference electrode was silver; all spectra were referenced against ferrocene. The scan rate was 0.2 V/s. The solvent was dichloromethane and the conducting salt was tetrabutylammonium hexafluorophosphate (TBA[PF_6_] 0.1 M).

The intensity data of **4** were collected on a Bruker Venture D8 diffractometer (Bruker, Billerica, MA, USA) at 100 K with Mo-Kα (0.7107 Å) radiation. All structures were solved by direct methods and refined based on F^2^ by use of the ShelX [42,43] program package as implemented in OLex2 1.2 [44]. All non-hydrogen atoms were refined using anisotropic displacement parameters. Hydrogen atoms attached to carbon atoms were included in geometrically calculated positions using a riding model. Crystal and refinement data are shown in Table S3. Figures were created using Diamond.

Crystallographic data for the structural analyses have been deposited in the Cambridge Crystallographic Data Centre. Copies of this information may be obtained free of charge from the Director, CCDC, 12 Union Road, Cambridge CB2 1EZ, U.K. (Fax: +44-1223-336033; e-mail: deposit@ccdc.cam.ac.uk or http://www.ccdc.cam.ac.uk).

Optimised equilibrium geometries were calculated on a DFT level with the Gaussian 16 revision A.03 [45] quantum software package for a single molecule in the gas phase using the PBE1PBE/6-311++G(2d,2p) [32,33,34,35,36,37] level of theory including empirical dispersion corrections according to Grimme’s D3 [38,39] method involving Becke–Johnson damping (GD3BJ). For the Sn atom, we used the Stuttgart/Dresden (SDD) pseudo-potential [40,41]. The orbital energies of HOMO and LUMO and their energy differences were calculated for these optimised molecules. Frequency analyses were performed in all cases to confirm the absence of imaginary frequencies and thus prove that the obtained geometries corresponded to minima on the potential energy surface. The isodensity value for the molecular orbital isosurface representations was set to 0.02 a.u. in all cases. Absorption data were calculated using time-dependent DFT (TD-DFT) levels on the optimised ground state geometries with the same functional and basis set as described above, i.e., TD-PBE1PBE-GD3BJ/6-311++G(2d,2p)//PBE1PBE-GD3BJ/6-311++G(2d,2p) using SDD pseudo-potentials for Sn [32,33,34,35,36,37,38,39,40,41]. 

Detailed information on all the materials and methods can be found in the ESI.

## 4. Conclusions

In conclusion, we synthesised and fully characterised the new spirostannole **4**. Unusually, our DFT calculations showed that there is no lobe on the Sn in the LUMO, which means the Sn is not involved in the LUMO. Nevertheless, the HOMO-LUMO energy gap is low and the optical properties agree with previous results. Cyclic voltammetry measurements showed a quasi-reversible process with three redox pairs.

We also investigated the reaction of this spirostannole with methyllithium in NMR experiments and studied the molecular structure and optoelectronic properties of the reaction product **5** by DFT and TD-DFT calculations. The symmetry of the molecule was broken and the localisation of the orbitals changed in comparison to those of stannole **4**. The HOMO-LUMO energy gap of stannolate **5** is narrower than that of spirostannole **4** and the distribution of the transitions is different.

## Figures and Tables

**Table 1:** Values of E_pa_, E_pc_, E_1/2_ and |∆E_p_| against F/Fc^+^.

E_pa_ (mV)	E_pc_ (mV)	E_1/2_ (mV)	|∆E_p_|= |E_pc_-E_pa_| (mV)
93.0	2.9	48.0	90.1
359.9	244.3	302.1	115.6
556.4	440.0	498.2	116.4

## Supplementary Materials

The following are available online, the Supplementary Materials are Materials and Methods, Experimental Procedures, Analytical Data, Images, CIF Files, and XYZ Files.
