# Supplementary material for: Experimental and Theoretical Studies of a Spirostannole and Formation of a Pentaorganostannate
Source: Molecules. 2020 Oct 28;25(21):4993. doi: 10.3390/molecules25214993 (PMC7672547; doi:10.3390/molecules25214993)
Supplement: Supplementary file 1 [file molecules-25-04993-s001.zip › molecules-970315-sup/Supporting Information_Ramirez-y-Medina.pdf]

Supporting Information

Experimental and Theoretical Studies of a Spirostannole and Formation of a Pentaorganostannate †

Isabel-Maria Ramirez y Medina [1,2], Markus Rohdenburg [1,3], Waldemar Kipke [1,4], Enno Lork [1,5] and Anne Staubitz [1,6,*]

1 MAPEX Center for Materials and Processes, University of Bremen, Bibliothek Str. 1, 28359 Bremen, Germany

2 Institute for Organic and Analytical Chemistry, University of Bremen, Leobener Str. 7, 28359 Bremen, Germany; ramirez-y-medina@uni-bremen.de

3 Institute for Applied and Physical Chemistry, University of Bremen, Leobener Str. 5, 28359 Bremen, Germany; m.rohdenburg@uni-bremen.de

4 Institute for Organic and Analytical Chemistry, University of Bremen, Leobener Str. 7, 28359 Bremen, Germany; kipke@uni-bremen.de

5 Institute for Inorganic Chemistry and Crystallography, University of Bremen, Leobener Str. 7, 28359 Bremen, Germany; elo@uni-bremen.de

6 Institute for Organic and Analytical Chemistry, University of Bremen, Leobener Str. 7, 28359 Bremen, Germany

* Correspondence: staubitz@uni-bremen.de

† In memory of Hans J. Reich

Table of Contents

1 General Considerations 2

1.1 Abbreviations 2

1.2 Chemicals and Solvents 4

1.3 Analytical Instruments 4

1.4 Crystallography 5

2. Syntheses 6

2.1 2-Iodo-5-methylthiophene (**1**)[3] 6

2.2 1,8-Bis(5-methyl-thiophen-2-yl)octa-1,7-diyne (**2**) 6

2.3 1,1',3,3'-Tetrakis(5-methylthiophen-2-yl)-4,4',5,5',6,6',7,7'-octahydro-2,2'-spirobi[benzo[*c*]stannole] (**4**) 7

2.4 Monitoring of the Reaction of Spirostannole **4** with MeLi by NMR measurements – Lithium Pentaorganostannate **5**[5] 7

3. Crystallography 10

4. DFT and TD-DFT Calculations 13

4. Optical Properties and Cyclic Voltammetry Measurements 23

5. NMR Spectra 27

6. Literature 34

1. General Considerations

1.1 Abbreviations

calcd.	calculated
Cu(I)Cl	copper(I) chloride
Cu(I)I	copper(I) iodide
DCM	dichloromethane
DFT	density functional theory
EI	electron impact
Et_2O	diethyl ether
EtOAc	ethyl acetate
EtOH	ethanol
GB	glove box
HOMO	highest occupied molecular orbital
IR	infrared spectroscopy
LUMO	lowest unoccupied molecular orbital
MCP-PMT detector	micro-channel plate (MCP)-photomultiplier tubes (PMT) detector
MeOH	methanol
NIS	*N*-iodosuccinimide
NMR	nuclear magnetic resonance
$[PdCl_2(PPh_3)_2]$	bis(triphenylphosphine)palladium(II) dichloride
PFK	perfluorokerosene
ppm	parts per million
R	resolution (for mass spectrometry)

sat. soln.	saturated solution
SPS	solvent purification system
TCSPC	time-correlated single photon counting
TEA	triethylamine
THF	tetrahydrofuran
TLC	thin layer chromatography
TMS	tetramethylsilane

1.2 Chemicals and Solvents

Reactions under inert conditions were carried out using standard Schlenk techniques under a dry, inert N_2 or Ar atmosphere or in a N_2-filled glove box from Inert. All anhydrous solvents were taken from the solvent purification system (SPS), degassed by four freeze-pump-thaw cycles and stored under a N_2 atmosphere. All chemicals were commercially available and were used without further purification unless noted otherwise.

Table S1. List of supplier and purity of used chemicals.

Reagent	Supplier	Purity	Comments
Ammonium chloride	Carl Roth	≥ 99.5%	
Copper(I) chloride	Alfa Aesar	99.999%	stored in a GB
Copper(I) iodide	Alfa Aesar	99.998%	stored in a GB
N-Iodosuccinimide	Sigma-Aldrich	98%	
Magnesium sulfate	Grüssing	99%	
Methyllithium	Acros Organics		1.6 M in diethyl ether
2-Methylthiophene	Sigma-Aldrich	98%	
1,7-Octadiyne	VWR Chemicals	98%	
$[PdCl_2(PPh_3)_2]$	Strem Chemicals	99.9+%	
Sodium carbonate	Sigma-Aldrich	99.5%	
Sodium thiosulfate	Grüssing	97%	
Tin(IV) chloride	Sigma Aldrich		1 M in heptanes under N_2 atmosphere
p-Toluenesulfonic acid·H_2O	Acros Organics	97.5 %	

Table S2. List of suppliers and purity of used solvents.

Solvent	Comments
Dichloromethane	VWR Chemicals; for HPLC; anhydrous from the SPS
Diethyl ether	VWR Chemicals; ACS, Reag. Ph. Eur.
Ethyl acetate	VWR Chemicals; ACS, Reag. Ph. Eur.
Ethanol	VWR Chemicals; Ethanol absolute ≥99.8%
n-Pentane	VWR Chemicals; ACS, Reag. Ph. Eur.
Triethylamine	Sigma Aldrich; 99%; anhydrous; degassed
Tetrahydrofuran	VWR Chemicals; for HPLC; anhydrous from the SPS and degassed
Toluene	VWR Chemicals; for HPLC; anhydrous, from the SPS and degassed

1.3 Analytical Instruments

^{1}H, ^{13}C{^{1}H}, ^{7}Li and ^{119}Sn{^{1}H} NMR spectra were recorded on a *Bruke*r Avance Neo 600 or *Bruke*r DRX 500 at 25°C unless noted otherwise. All ^{1}H NMR and ^{13}C{^{1}H} NMR spectra were referenced against the solvent residual proton signals (^{1}H), or the solvent itself (^{13}C). The reference for the ^{7}Li and ^{119}Sn{^{1}H} NMR spectra was calculated based on the ^{1}H NMR spectrum of TMS. All chemical δ shifts are given in parts per million (ppm) and all coupling constants J in Hz. The following abbreviations are used to describe splitting patterns: br s = broad singlet, s = singlet, d = doublet, dd = doublet of doublets, m = multiplet.

Electron Impact (EI) ionisation mass spectra were obtained on the double focusing mass spectrometer MAT 95XL. Samples were measured by direct inlet or indirect inlet method with a source temperature of 200° C. The ionisation energy of the electron impact ionisation was 70 eV. All signals were reported with the quotient from mass to charge *m*/*z*. High resolution (HR) EI mass

spectra were recorded on the double focusing mass spectrometer MAT 95XL from FINNIGAN MAT. Precision weights were determined via the peak-matching method. The reference substance was perfluorokerosene (PFK). The resolution (R) of the peak-matching performance was 10000. The calculated isotopic distribution for each ion agreed with experimental values.

IR spectra were recorded on a *Nicolet* Thermo IS10 SCIENTIFIC spectrometer with a diamond ATR unit. The resolution was 4 cm^{-1}. Relative intensities of the IR bands were described by s = strong (0 – 33% T), m = medium (34 – 66% T) or w = weak (67 – 100% T).

All melting points were measured with a *Büchi* Melting Point M-560 apparatus and are uncorrected.

Thin layer chromatography (TLC) was carried out on aluminum plates from Macherey-Nagel that were coated with silica gel 60 F_{254} with a layer thickness of 0.2 mm. All bands were detected by using UV light (254 nm and 366 nm). Column chromatography was carried out by using the column machine PuriFlash 4250 from Interchim. Silica gel columns of the type PF-15SiHP-F0025 and PF-50SiHP-JP-F0120 were used. The injection of the sample was performed via dry load. The column material of the dry load was Celite 503 from Macherey-Nagel.

UV/Vis spectra were recorded with a resolution of 0.1 nm on a UV-2700 spectrometer from *Shimadzu*.

Emission spectra were recorded on an Edinburgh Instruments FLS 1000 photoluminescence spectrometer. Absolute quantum yields (QY) were measured with an Edinburgh Instruments integrating sphere. TCSPC measurements were performed using a fast response MCP-PMT detector and a 376 nm Edinburgh Instruments EPL Laser as excitation source with 10 – 20 MHz repetition rate and 80 ps pulse width. Temperature dependent-fluorescence measurements were carried out using an Oxford Instruments Optistat-CF cryostat cooled with liquid nitrogen. All emission spectra are corrected spectra.

Cyclic voltammetry measurements were carried out using the potentiostat Autolab PGSTAT101 Metrohm. The material of the working electrode and the counter electrode was platinum. The material of the reference electrode was silver; all spectra were referenced against ferrocene. The scan rate was 0.2 V/s. The solvent was dichloromethane and the conducting salt was tetrabutylammonium hexafluorophosphate (TBA[PF_6] 0.1 M).

1.4 Crystallography

Intensity data of **4** was collected on a Bruker Venture D8 diffractometer at 100 K with Mo-Kα (0.7107 Å) radiation. All structures were solved by direct methods and refined based on F^2 by use of the SHELX[1-2] program package as implemented in OLex2 1.2.[3] All non-hydrogen atoms were refined using anisotropic displacement parameters. Hydrogen atoms attached to carbon atoms were included in geometrically calculated positions using a riding model. Crystal and refinement data are collected in Table S3. Figures were created using DIAMOND.

Crystallographic data for the structural analyses have been deposited within Cambridge Crystallographic Data Centre. Copies of this information may be obtained free of charge from the Director, CCDC, 12 Union Road, Cambridge CB2 1EZ, UK (Fax: +44-1223-336033; e-mail: deposit@ccdc.cam.ac.uk or http://www.ccdc.cam.ac.uk).

2. Syntheses

2.1 2-Iodo-5-methylthiophene (1)[4]

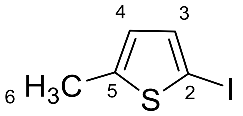

This reaction was carried out under normal atmosphere. To a solution of 2-methylthiophene (5.00 g, 50.9 mmol) in EtOH (70 mL), NIS (12.6 g, 56.0 mmol) and *p*-toluenesulfonic acid (970 mg, 5.09 mmol, 10 mol%) were added at 22 °C. The reaction mixture was stirred at 22 °C for 10 min and a sat. soln. of $Na_2S_2O_3$ (88 mL) was added. It was extracted with EtOAc (3 x 100 mL) and the organic phase was separated. It was washed with a 1 M solution of Na_2CO_3 (100 mL), dried over $MgSO_4$, filtered, and dried *in vacuo*. Purification by Kugelrohr distillation (85 °C, 11 mbar) gave the product as a light yellow oil (11.4 g, 61%). ^{1}H NMR (500 MHz, $CDCl_3$): δ 7.03 (d, $^3J_{HH}$ = 3.5 Hz, 1H, H-3), 6.46 (d, $^3J_{HH}$ = 3.5 Hz, 1H, H-4), 2.48 (s, 3H, H-6) ppm; ^{13}C{^{1}H} NMR (126 MHz, $CDCl_3$): δ 146.1 (C-5), 136.9 (C-3), 127.1 (C-4), 69.5 (C-2), 15.5 (C-6) ppm; IR (ATR): *ν* 2917 (w), 2855 (w), 1538 (w), 1432 (m), 1213 (m), 1158 (m), 1050 (w), 982 (w), 929 (m), 786 (s), 737 (w), 695 (w), 664 (m) cm^{-1}; HR-MS (EI, C_5H_5IS) calcd. 223.91512, found 223.91511 (R = 10000); MS (EI, 70 eV, indirect inlet, 200 °C): *m*/*z* (% relative intensity) = 224 (100) $[M]^{+\cdot}$.

The analytical data are in agreement with the literature.[4]

2.2 1,8-Bis(5-methyl-thiophen-2-yl)octa-1,7-diyne (2)

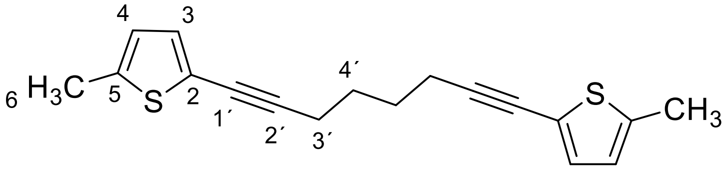

In a N_2-filled GB, a mixture of 2-methyl-5-iodothiophene (2.00 g, 8.93 mmol), $[PdCl_2(PPh_3)_2]$ (253 mg, 4 mol%, 360 μmol) and Cu(I)I (68.6 mg, 4 mol%, 360 μmol) in anhydrous, degassed TEA (5.00 mL) and anhydrous, degassed toluene (5.00 mL) was stirred at 22 °C. After addition of 1,7-octadiyne (450 mg, 4.24 mmol), the suspension was stirred at 80 °C for 8 h.

Then, the reaction was quenched with a sat. soln. of NH_4Cl (1 x 50 mL). The aqueous layer was extracted with Et_2O (3 x 100 mL) and the combined organic layers were washed with H_2O (1 x 100 mL). The phases were separated, the organic phase was dried over $MgSO_4$ and filtered. The volatiles were removed *in vacuo*. The product was purified by column chromatography (gradient from 100% *n*-pentane to 1:5 DCM/*n*-pentane, R_f = 0.44) to give a yellow-brownish oil, which crystallised at 5 °C (527 mg, 40%). M.P.: 50 °C; ^{1}H NMR (500 MHz, $CDCl_3$): δ 6.92 (d, $^3J_{HH}$ = 3.5 Hz, 2H, H-3), 6.58 (d, $^3J_{HH}$ = 3.5 Hz, $^4J_{HH}$ = 1.1 Hz, 2H, H-4), 2.52 – 2.45 (m, 4H, H-3´), 2.44 (s, 6H, H-6), 1.77 – 1.72 (m, 4H, 4´) ppm; ^{13}C{^{1}H} NMR (126 MHz, $CDCl_3$): δ 140.7 (C-5), 131.3 (C-3), 125.1 (C-4), 121.7 (C-2), 93.0 (C-2´), 74.5 (C-1´), 27.9 (C-3´), 19.4 (C-4´), 15.5 (C-6) ppm; IR (ATR): *ν* 2952 (w), 2932 (w), 2895 (w), 2866 (w), 2821 (w), 1751 (w), 1605 (w), 1538 (w), 1470 (m), 1457 (w), 1418 (m), 1333 (w), 1320 (w), 1306 (w), 1236 (w), 1187 (m), 1159 (m), 1045 (m), 992 (w), 966 (w), 898 (w), 888 (m), 875 (w), 801 (s), 758 (m), 732 (w), 675 (m) cm^{-1}; HR-MS (EI, $C_{18}H_{18}S_2$) calcd. 298.08444, found 298.08456 (R = 10000); MS (EI, 70 eV, direct inlet, 200 °C): *m*/*z* (% relative intensity) = 298 (100) $[M]^{+\cdot}$.

Rosenthal´s zirconocene (**3**) was reproduced according to the known procedure of Rosenthal et al.[5-6]

2.3 1,1',3,3'-Tetrakis(5-methylthiophen-2-yl)-4,4',5,5',6,6',7,7'-octahydro-2,2'-spirobi[benzo[*c*]stannole] (4)

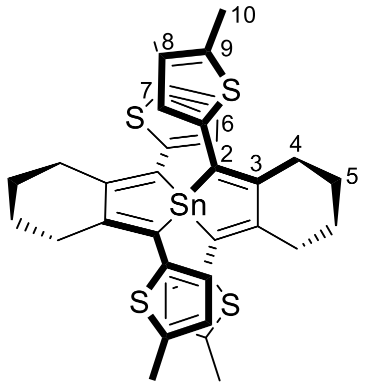

In a N_2-filled GB, 1,8-bis(5-methylthiophen-2-yl)octa-1,7-diyne (150 mg, 503 µmol) and Rosenthal´s zirconocene (237 mg, 503 µmol) were dissolved in anhydrous, degassed toluene (8 mL). The dark red solution was stirred at 22 °C for 18 h. Then, Cu(I)Cl (4.9 mg, 50 µmol) was added. At the Schlenk line, the reaction mixture was stirred at -78 °C and $SnCl_4$ (0.25 mL, 252 µmol, 1 M in heptanes) was added dropwise over the course of 1 min. The reaction was stirred at -78 °C for 1 h and then at 22 °C for 16 h. The reaction mixture was quenched with H_2O (1 x 50 mL) and extracted with Et_2O (3 x 50 mL). The combined organic layers were dried over $MgSO_4$, filtered and concentrated *in vacuo*. The crude product was purified by column chromatography (silica gel, gradient from 100% *n*-pentane to 100% DCM, R_f = 0.14) and then crystallised from a sat. soln. of toluene to give orange-yellow crystals (50 mg, 28%). M.P.: the colour of the compound changed from orange-yellow to dark reddish-black at 232 °C and melted at 248 °C; $\lambda_{abs, max}$ ($CHCl_3$, ε = 31235 L·mol^{-1}·cm^{-1}) = 436 nm; $\lambda_{em, max}$ ($CHCl_3$) = 533 nm; ^{1}H NMR (600 MHz, $CDCl_3$): δ 6.60 (d, $^3J_{HH}$ = 3.6 Hz, 2H, H-7), 6.52 (d, $^3J_{HH}$ = 3.6 Hz, $^4J_{HH}$ = 1.2 Hz, 2H, H-8), 2.95 – 2.82 (m, 4H, H-4), 2.40 (s, 6H, H-10), 1.86 – 1.78 (m, 4H, 5) ppm; ^{13}C{^{1}H} NMR (151 MHz, $CDCl_3$): δ 147.1 (C-3, $^2J_{SnC}$ = 42.7 Hz), 143.1 (C-6, $^2J_{SnC}$ = 39.0 Hz), 140.6 (C-9), 129.9 (C-7, $^4J_{SnC}$ = 14.5 Hz), 129.3 (C-2, $^1J_{SnC}$ = 226.5, 217 Hz), 125.5 (C-8), 31.6 (C-4, $^3J_{SnC}$ = 31.2 Hz), 23.4 (C-5), 15.7 (C-10) ppm; ^{119}Sn{^{1}H} NMR (224 MHz, $CDCl_3$): δ -36.0 ppm; IR (ATR): ν 3075 (w), 2946 (w), 2911 (w), 2851 (w), 2814 (w), 1524 (w), 1432 (w), 1415 (w), 1371 (w), 1358 (w), 1338 (w), 1250 (w), 1211 (m), 1158 (w), 1133 (w), 1088 (w), 1052 (w), 1021 (w), 992 (w), 922 (w), 901 (w), 863 (w), 822 (w), 788 (m), 738 (w), 713 (w), 661 (m) cm^{-1}; HR-MS (EI, $C_{18}H_{18}S_2$) calcd. 712.07195, found 712.07069 (R = 10000); MS (EI, 70 eV, direct inlet, 200 °C): *m*/*z* (% relative intensity) = 716 (100) [M]$^{+\cdot}$.

2.4 Monitoring of the Reaction of Spirostannole 4 with MeLi by NMR measurements – Lithium Pentaorganostannate 5[7-8]

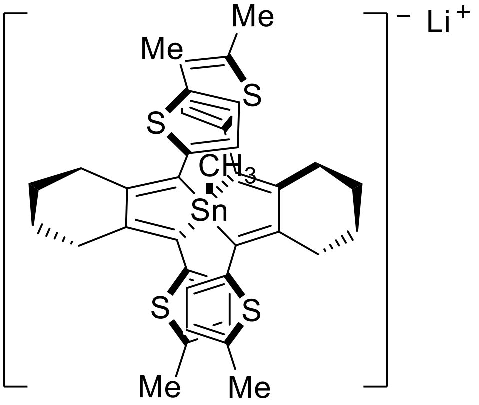

In an inert NMR tube, spirostannole **4** (5.8 mg, 8.1 µmol) was dissolved in anhydrous, degassed THF under an Ar atmosphere. At -78 °C, MeLi (1.6 M in Et_2O, 6 µL, 8.9 µmol) was added to this solution, the colour directly changed from orange-yellow to red. NMR spectra were recorded at -35 °C (this was the minimum temperature of the device) and then again at 25 °C. After finishing all measurements, the mixture was brownish-turbid yellow indicating the decomposition of the compound. $^{119}Sn\{^{1}H\}$ NMR (224 MHz, $CDCl_3$, 238.15 K): δ -211.2 ppm; ^{7}Li NMR (233 MHz, $CDCl_3$, 238.15 K): δ -0.4 ppm.

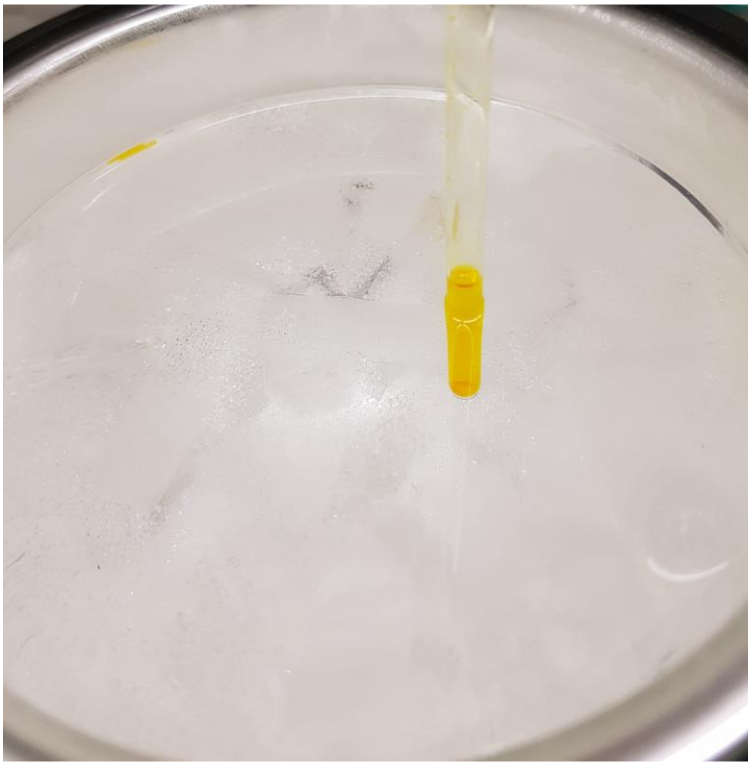

Figure S1. Spirostannole 4 in degassed, anhydrous THF at −78 °C in an inert NMR tube.

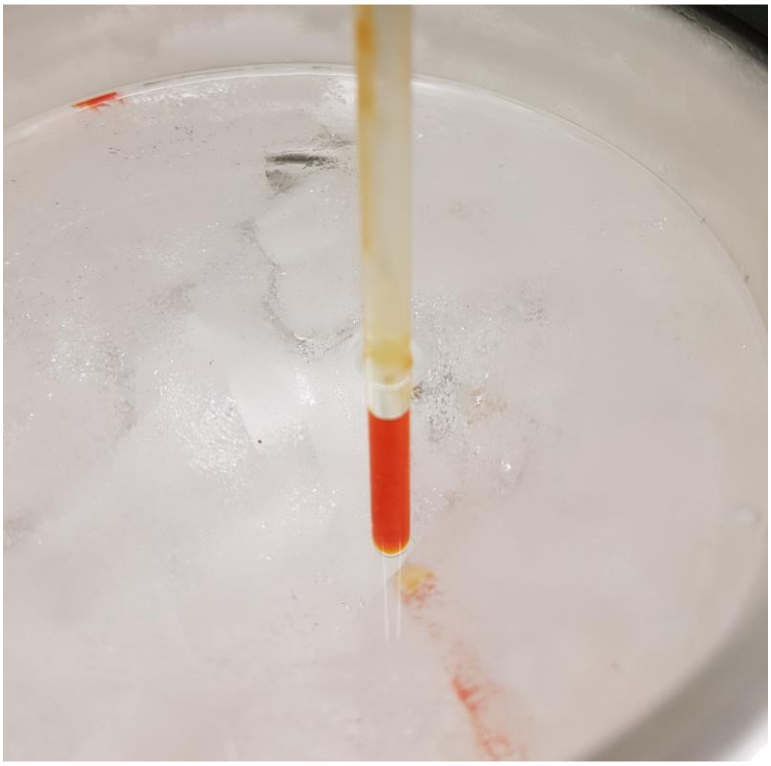

Figure S2. Spirostannole 4 with MeLi in degassed, anhydrous THF at −78 °C in an inert NMR tube.

3. Crystallography

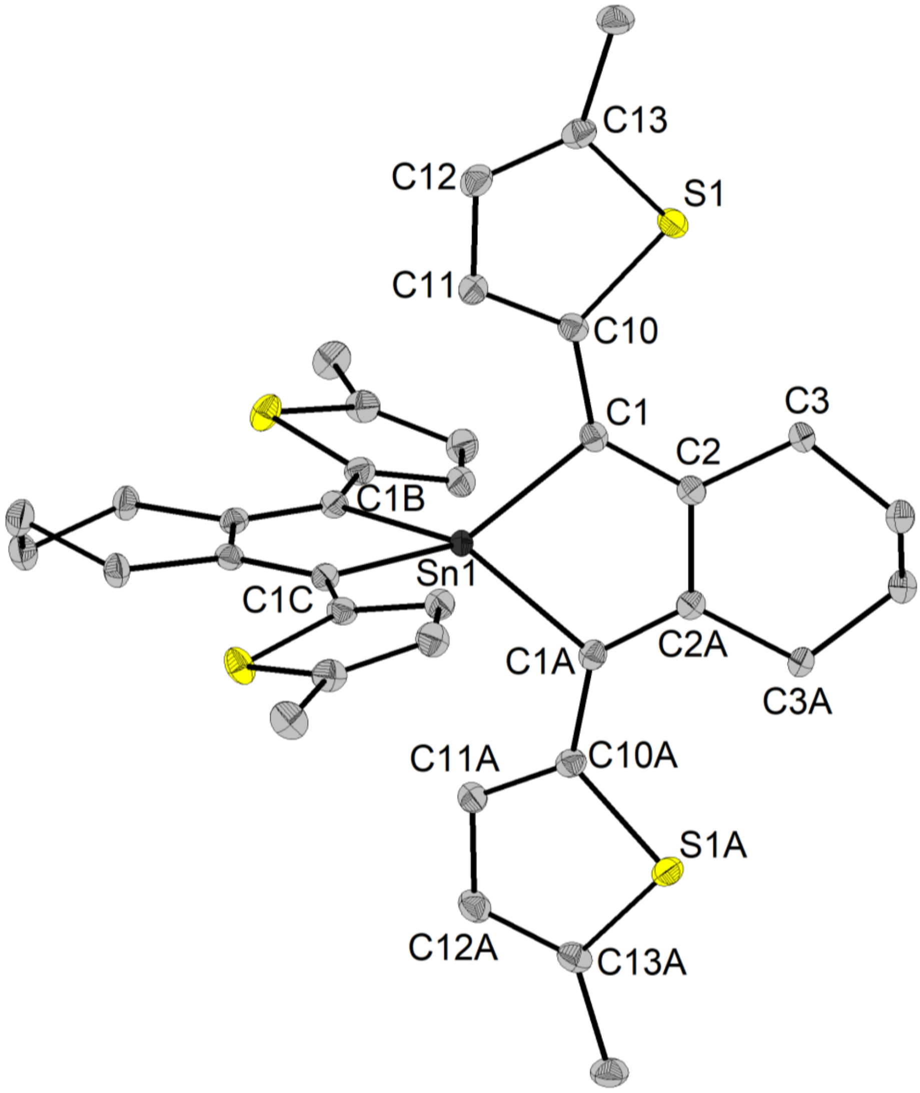

Figure S3. Molecular structure of spirostannole 4 showing 50% probability ellipsoids and the crystallographic numbering scheme. All hydrogen atoms are omitted for clarity.

Table S3. Crystal data and structure refinement for 4.

**CCDC number**	**2027202**
Empirical formula	$C_{36}H_{36}S_4Sn$
Formula weight	715.58
Temperature [K]	100.0
Crystal system	'orthorhombic'
Space group (number)	*Ccce* (68)
a [Å]	14.5502(7)
b [Å]	17.9138(9)
c [Å]	11.6528(6)
α [Å]	90
β [Å]	90
γ [Å]	90
Volume [Å^3]	3037.3(3)
Z	4

ρ_{calc} [g/cm^3]	1.565
μ [mm^{-1}]	1.142
F(000)	1464
Crystal size [mm^3]	0.2×0.2×0.1
Crystal colour	orange
Crystal shape	plate
Radiation	MoK_α (λ=0.71073 Å)
2θ range [°]	5.02 to 56.99 (0.74 Å)
Index ranges	-19 ≤ h ≤ 19<br>-24 ≤ k ≤ 23<br>-13 ≤ l ≤ 15
Reflections collected	14689
Independent reflections	1930<br>R_{int} = 0.0387<br>R_{sigma} = 0.0258
Completeness to θ = 25.242°	99.8 %
Data / Restraints / Parameters	1930/0/95
Goodness-of-fit on F^2	1.088
Final R indexes [I≥2σ(I)]	R_1 = 0.0285<br>wR_2 = 0.0703
Final R indexes [all data]	R_1 = 0.0360<br>wR_2 = 0.0743
Largest peak/hole [eÅ^3]	1.01/-0.47

Table S4. Selected bond lengths and angles for 4.

	**Length [Å]**
Sn1–C1^B	2.136(2)
Sn1–C1^A	2.136(2)
Sn1–C1	2.136(2)
Sn1–C1^C	2.136(2)
S1–C10	1.755(2)
S1–C13	1.725(2)
C1–C2	1.357(3)
C1–C10	1.452(3)
C11–C10	1.373(3)
C11–C12	1.419(3)
C2–C2^A	1.507(4)
C2–C3	1.520(3)
C3–C4	1.524(3)
C12–C13	1.364(3)
C4–C4^A	1.510(5)
C14–C13	1.510(3)
	**Angle [°]**
C1^B–Sn1–C1^C	83.5(2)
C1^A–Sn1–C1	83.5(2)
C1^B–Sn1–C1	126.0(2)
C1^B–Sn1–C1^A	121.6(2)
C1^C–Sn1–C1^A	126.0(2)
C1^C–Sn1–C1	121.6(2)
C13–S1–C10	93.2(2)
C2–C1–Sn1	108.6(2)
C2–C1–C10	129.7(3)
C10–C1–Sn1	121.6(2)
C10–C11–C12	114.5(2)
C1–C2–C2^A	119.5(2)

C1–C2–C3	121.5(2)
$C2^A$–C2–C3	118.9(2)
C2–C3–C4	115.1(2)
C1–C10–S1	125.5(3)
C11–C10–S1	108.4(2)
C11–C10–C1	126.0(2)
C13–C12–C11	113.4(2)
$C4^A$–C4–C3	109.6(3)
C12–C13–S1	110.3(3)
C12–C13–C14	129.2(2)
C14–C13–S1	120.4(2)

Symmetry transformations used to generate equivalent atoms: A: X, 0.5-Y, 0.5-Z; B: 1-X, Y, 0.5-Z; C: 1-X, 0.5-Y, Z;.

Table S5. Selected torsion angles for 4.

	**Torsion Angle [°]**
Sn1–C1–C2–$C2^A$	1.8(3)
Sn1–C1–C2–C3	-178.4(2)
Sn1–C1–C10–S1	-167.3(2)
Sn1–C1–C10–C11	13.7(3)
C1–C2–C3–C4	165.2(2)
C11–C12–C13–S1	-0.2(3)
C11–C12–C13–C14	179.2(2)
C2–C1–C10–S1	15.7(3)
C2–C1–C10–C11	-163.3(2)
$C2^A$–C2–C3–C4	-15.1(3)
C2–C3–C4–$C4^A$	49.0(3)
C10–S1–C13–C12	-0.4(3)
C10–S1–C13–C14	-179.9(3)
C10–C1–C2–$C2^A$	179.1(2)
C10–C1–C2–C3	-1.1(4)
C10–C11–C12–C13	1.0(3)
C12–C11–C10–S1	-1.3(2)
C12–C11–C10–C1	177.8(2)
C13–S1–C10–C1	-178.1(2)
C13–S1–C10–C11	1.0(2)

Symmetry transformations used to generate equivalent atoms: A: X, 0.5-Y, 0.5-Z;.

4. DFT and TD-DFT Calculations

Optimised equilibrium geometries were calculated on a DFT level with the Gaussian 16, revision A.03[9] quantum software package for a single molecule in the gas phase using the PBE1PBE/6-311++G(2d,2p)[10-15] level of theory including empirical dispersion corrections according to Grimme's D3[16-17] method involving Becke-Johnson damping (GD3BJ). For the Sn atom, we employed the Stuttgart/Dresden (SDD) pseudo potential.[18-19] The orbital energies of HOMO and LUMO and their energy differences were calculated for these optimised molecules. Frequency analyses were performed in all cases to confirm the absence of imaginary frequencies and thus prove that the obtained geometries corresponded to minima on the potential energy surface. The isodensity value for the molecular orbital isosurface representations was set to 0.02 a.u. in all cases. Absorption data was calculated using time-dependent DFT (TD-DFT) level on the optimised ground state geometries with the same functional and basis set as described above, i.e., TD-PBE1PBE-GD3BJ/6-311++G(2d,2p)//PBE1PBE-GD3BJ/6-311++G(2d,2p) employing SDD pseudo potentials for Sn.[10-19]

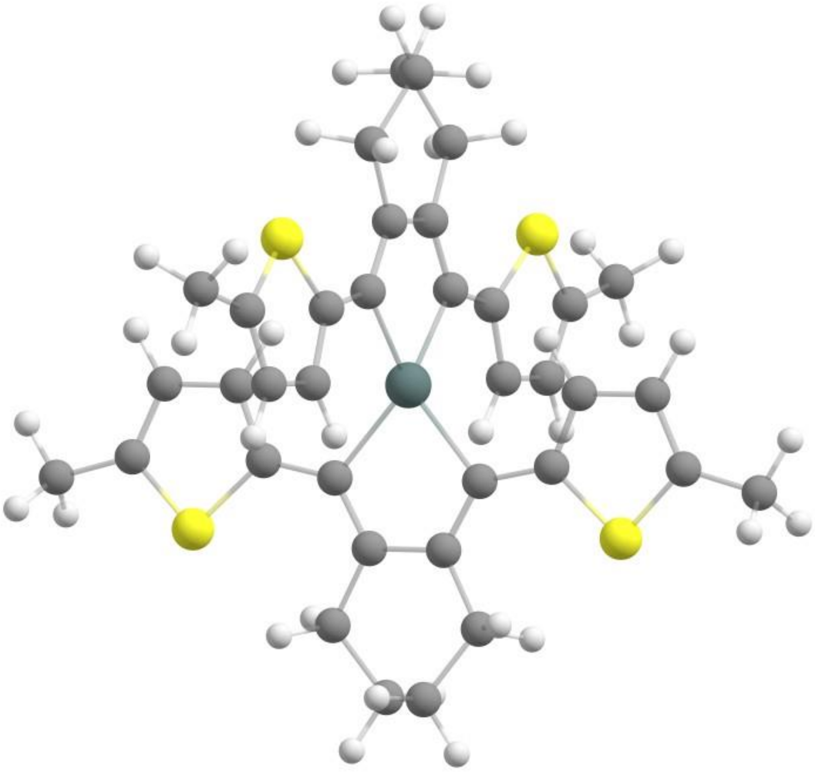

Figure S4. Optimised geometry of spirostannole **4**.

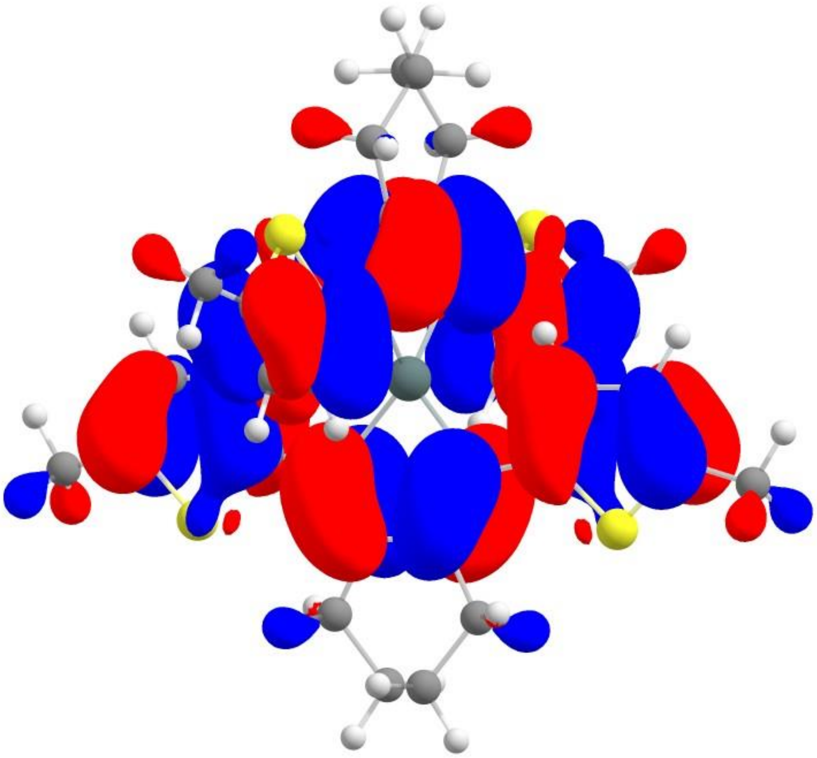

Figure S5. HOMO 1 of spirostannole 4.

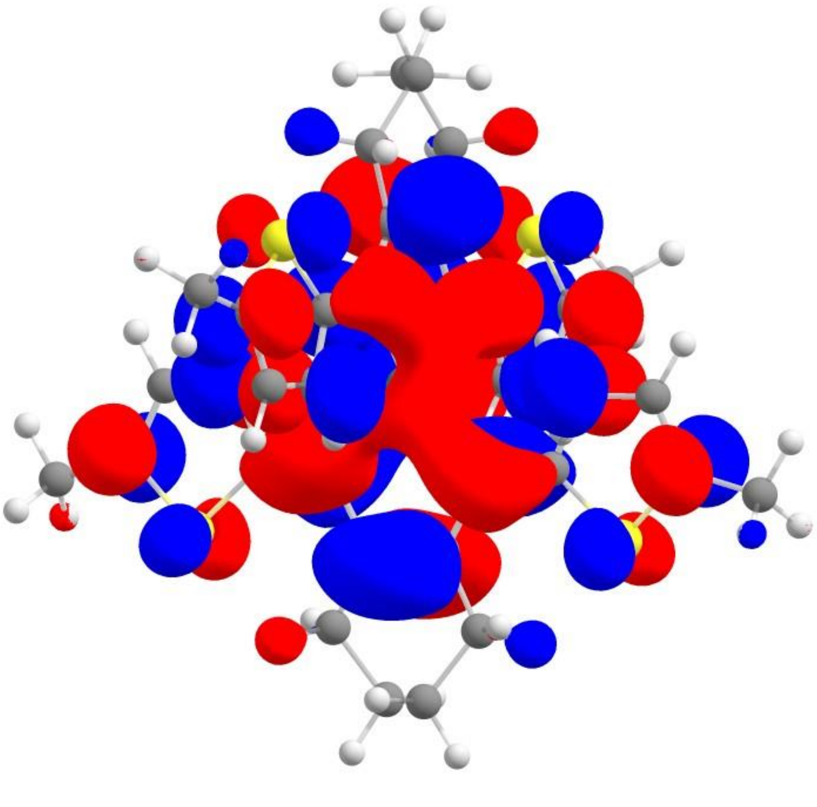

Figure S6. LUMO 1 of spirostannole 4.

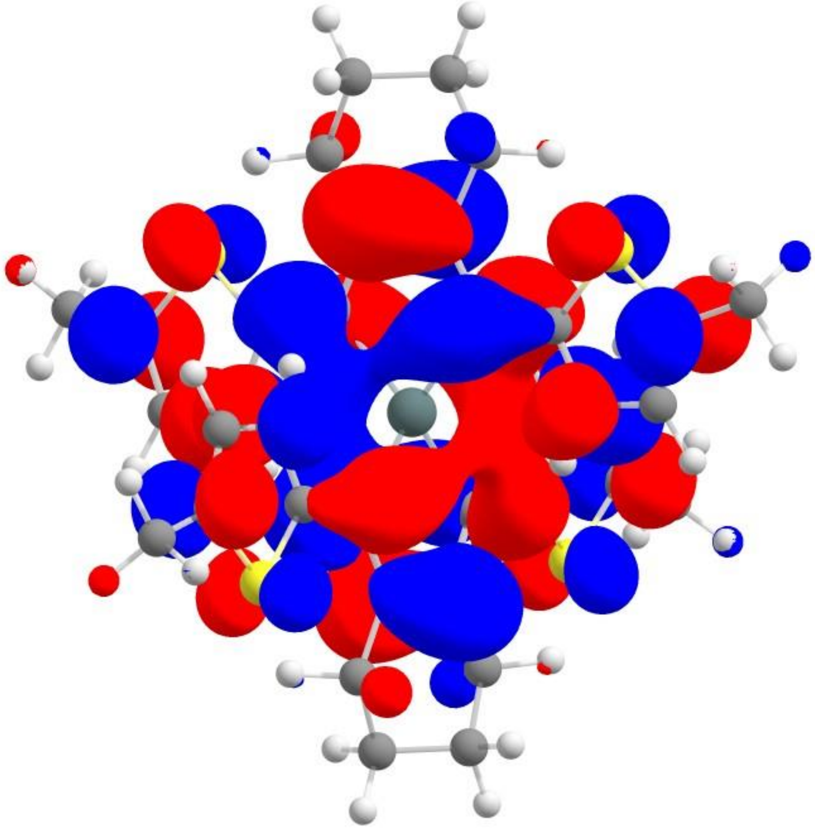

Figure S7. LUMO 1 of spirostannole 4, other viewing direction.

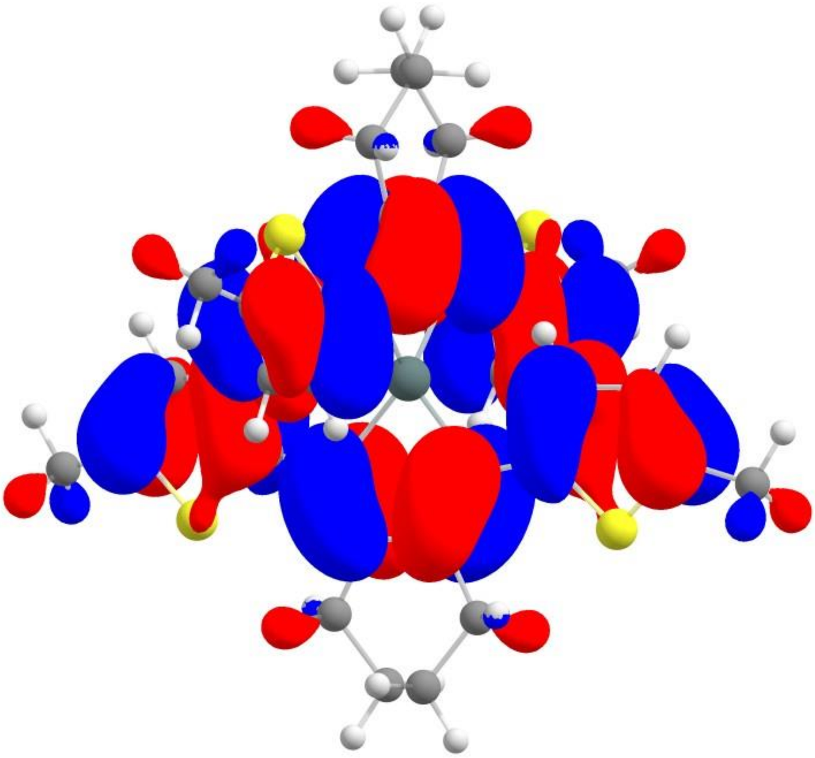

Figure S8. HOMO 2 of spirostannole 4.

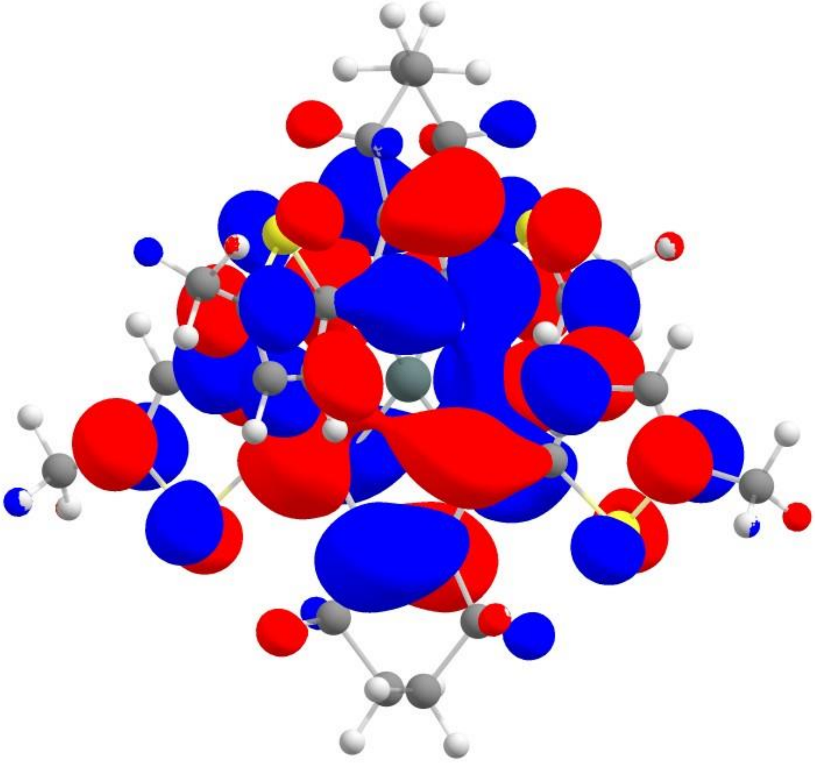

Figure S9. LUMO 2 of spirostannole 4.

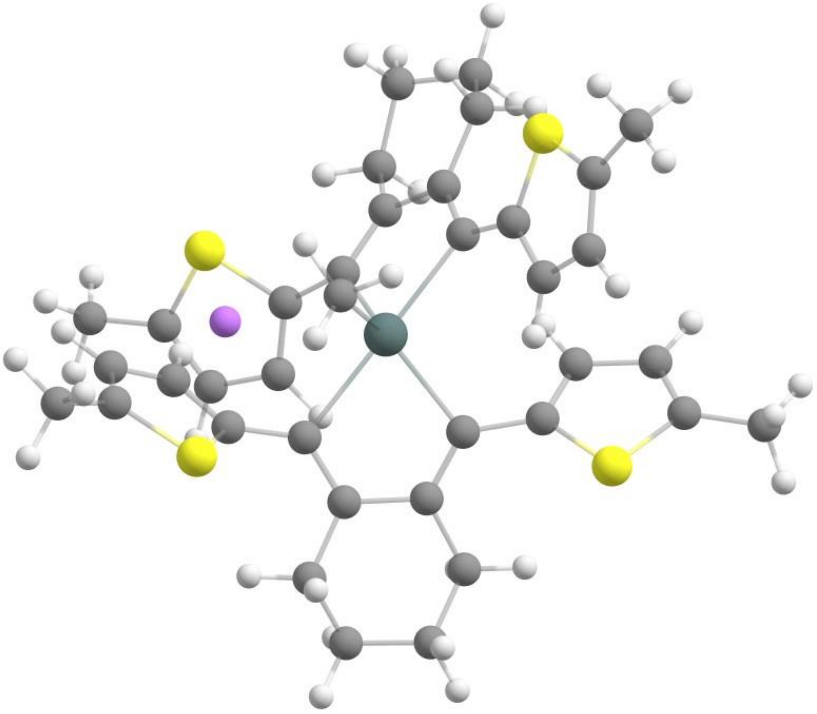

Figure S10. Optimised geometry of lithium pentaorganostannate 5.

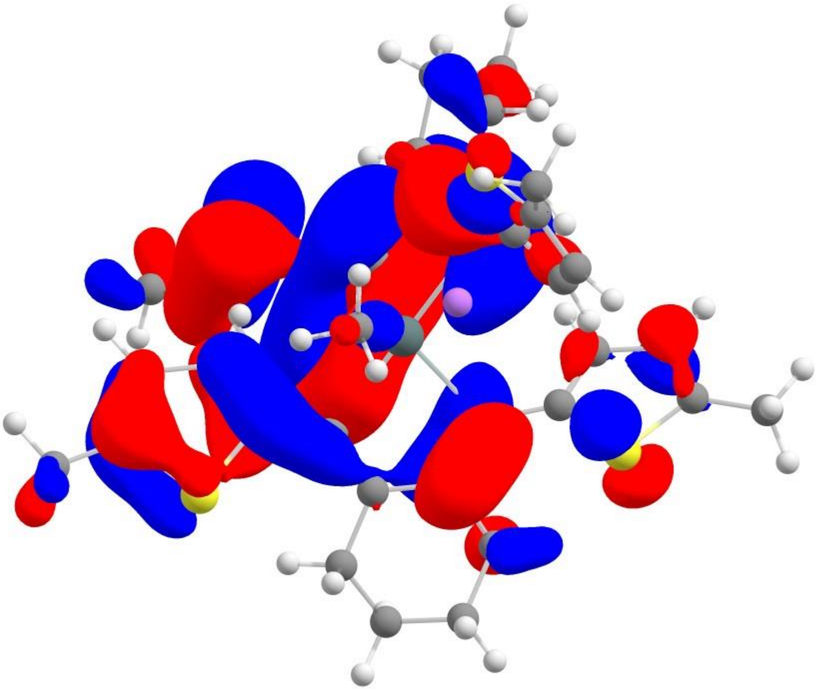

Figure S11. HOMO-4 of lithium pentaorganostannate 5.

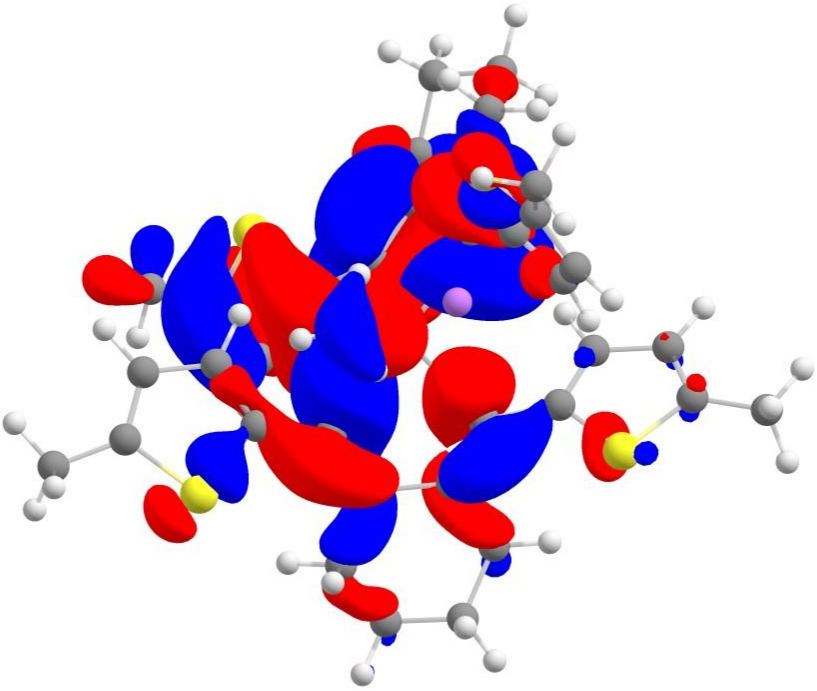

Figure S12. HOMO-2 of lithium pentaorganostannate 5.

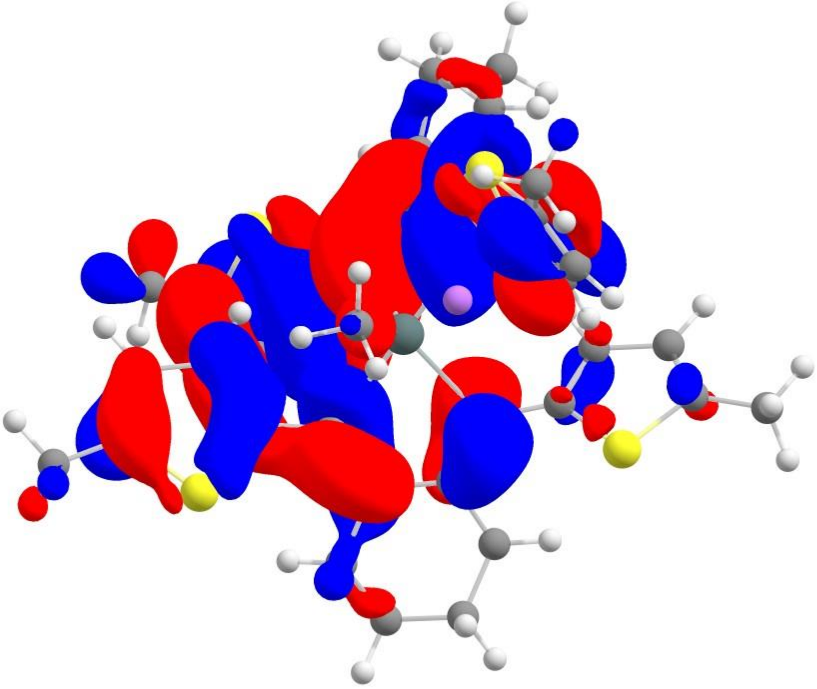

Figure S13. HOMO-1 of lithium pentaorganostannate 5.

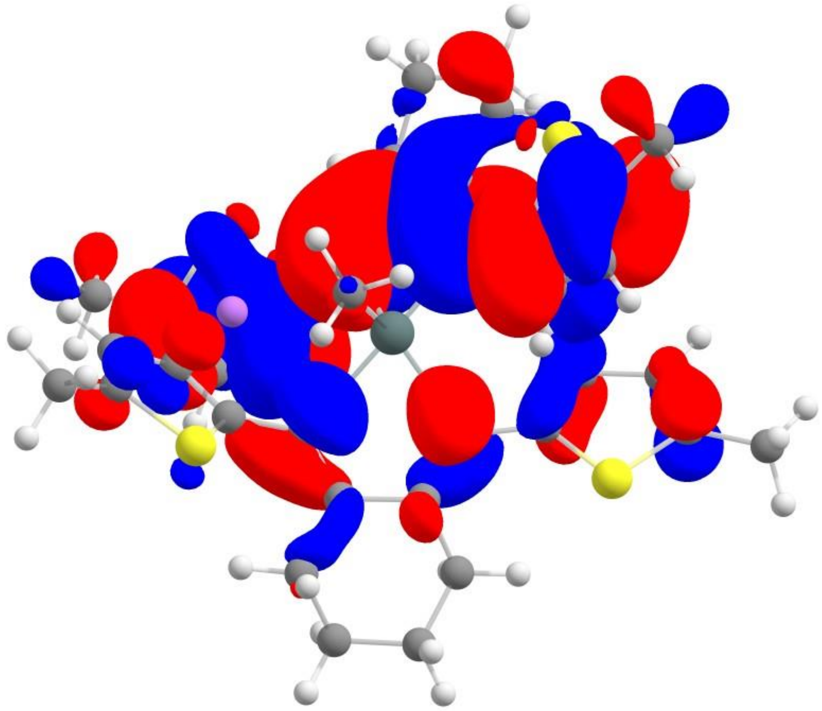

Figure S14. HOMO of lithium pentaorganostannate 5.

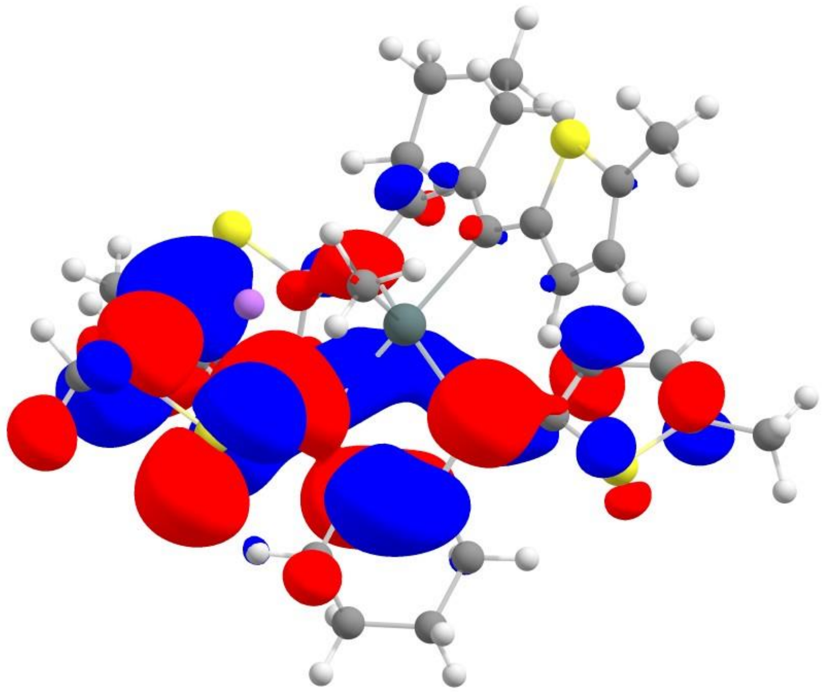

Figure S15. LUMO of lithium pentaorganostannate 5.

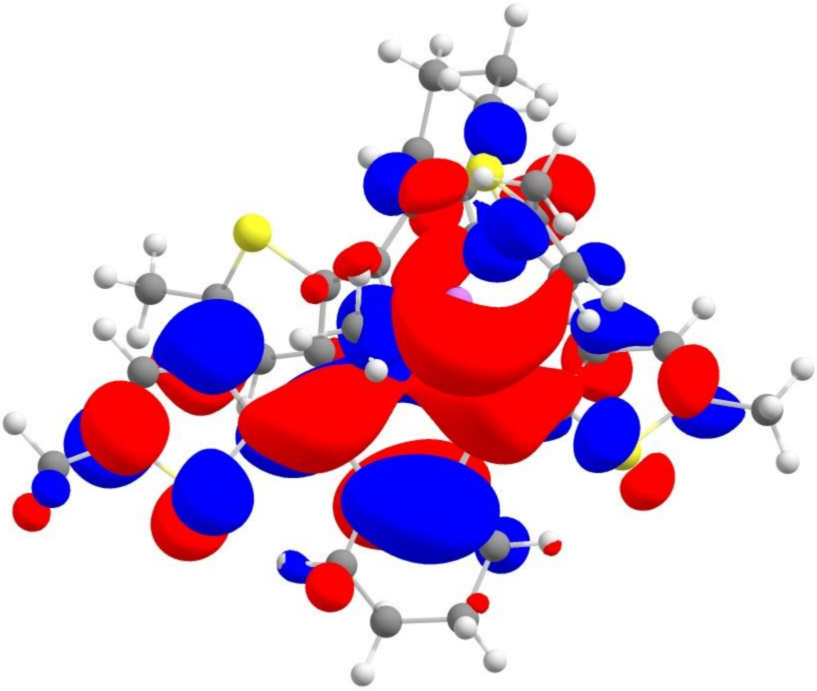

Figure S16. LUMO+1 of lithium pentaorganostannate 5.

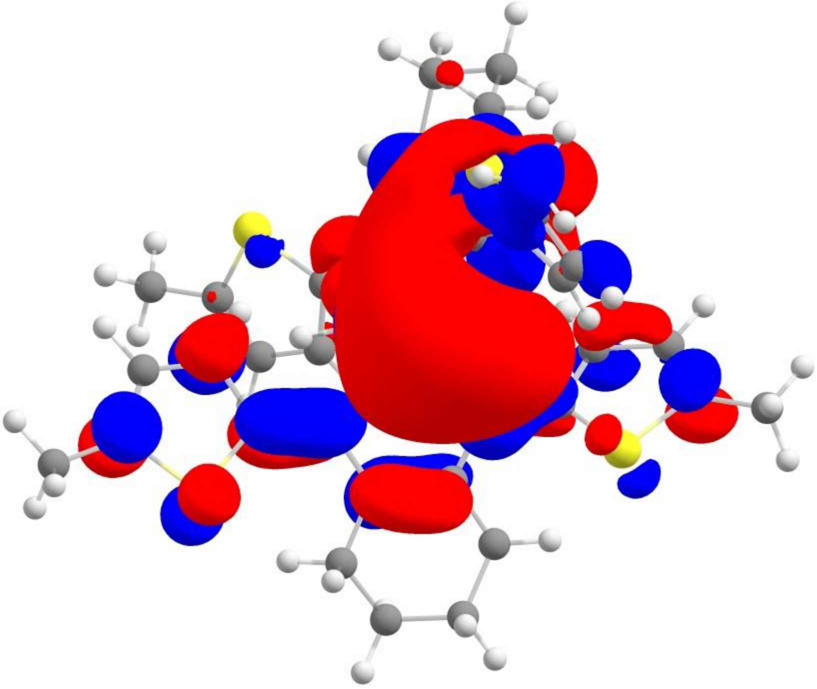

Figure S17. LUMO+2 of lithium pentaorganostannate 5.

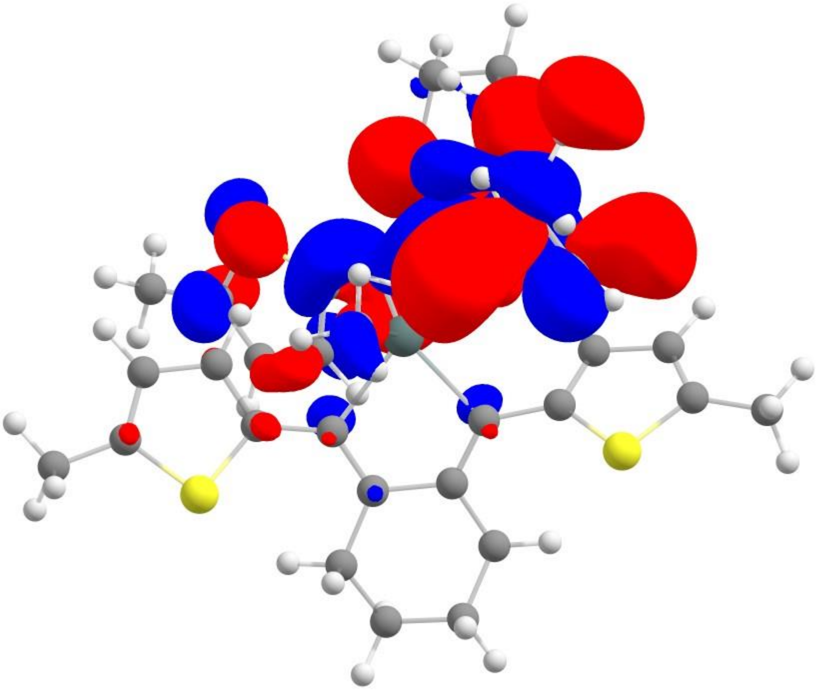

Figure S18. LUMO+3 of lithium pentaorganostannate 5.

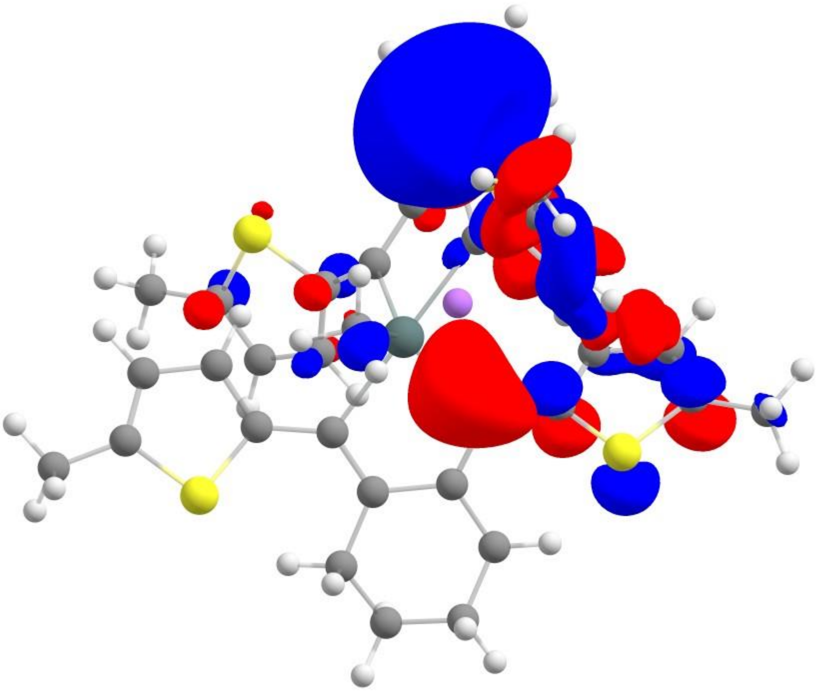

Figure S19. LUMO+4 of lithium pentaorganostannate 5.

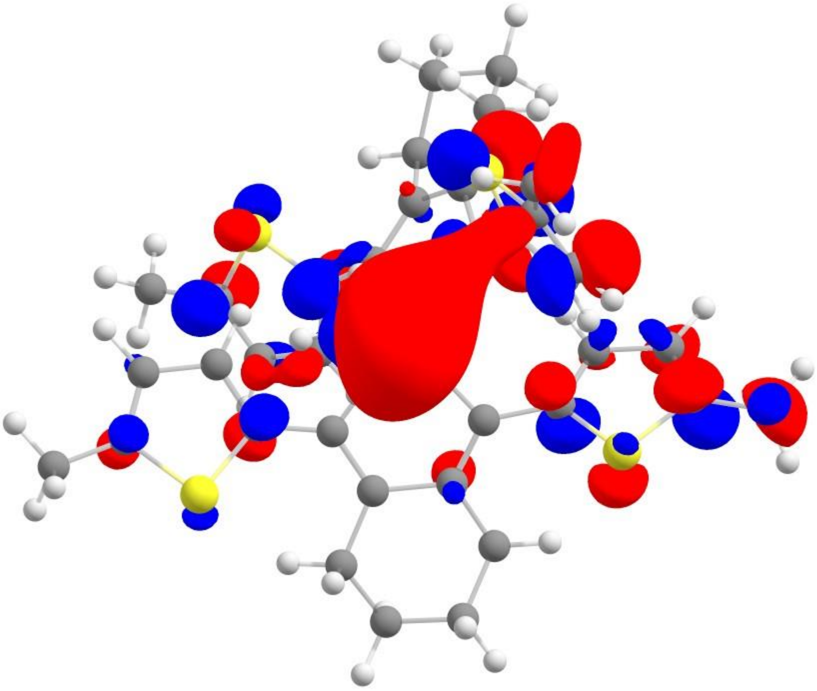

Figure S20. LUMO+5 of lithium pentaorganostannate 5.

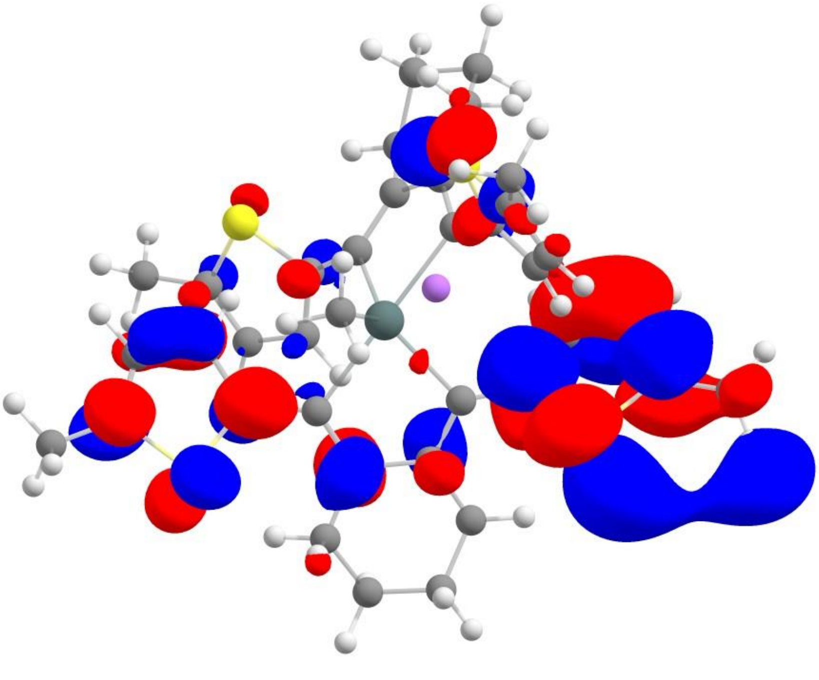

Figure S21. LUMO+7 of lithium pentaorganostannate 5.

4. Optical Properties and Cyclic Voltammetry Measurements

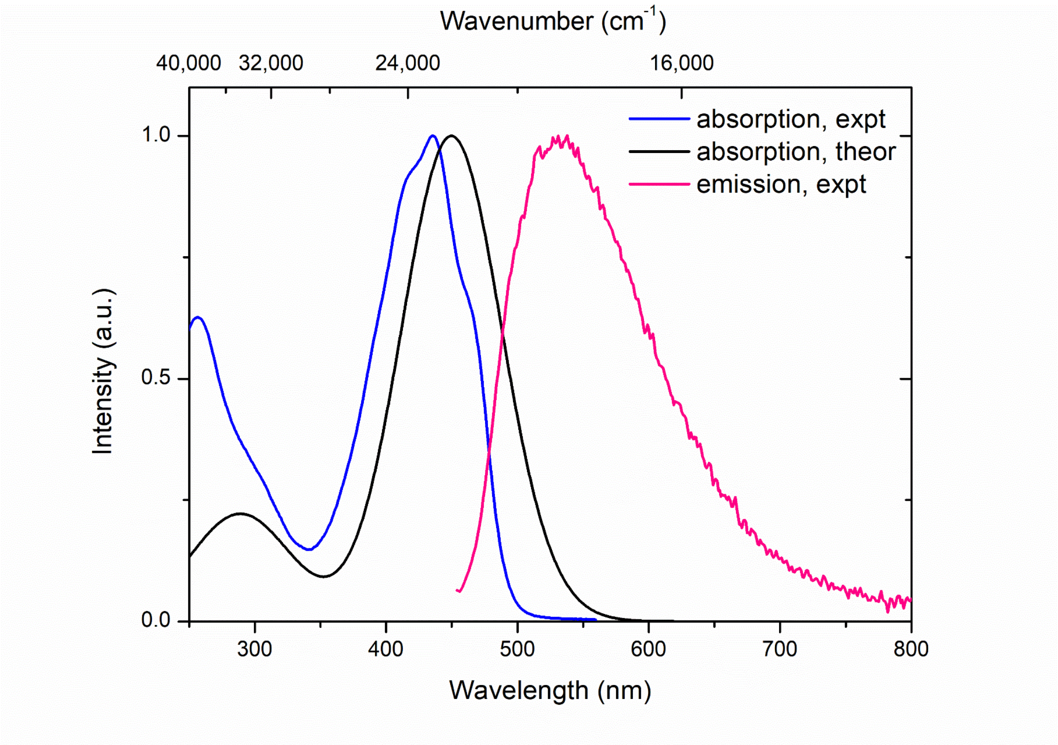

Figure S22. Normalised absorption and emission spectra of stannole 4 in chloroform (c = 10^{-5} mol L^{-1}) and normalised theoretical absorption spectrum. The emission was monitored by excitation at 445 nm.

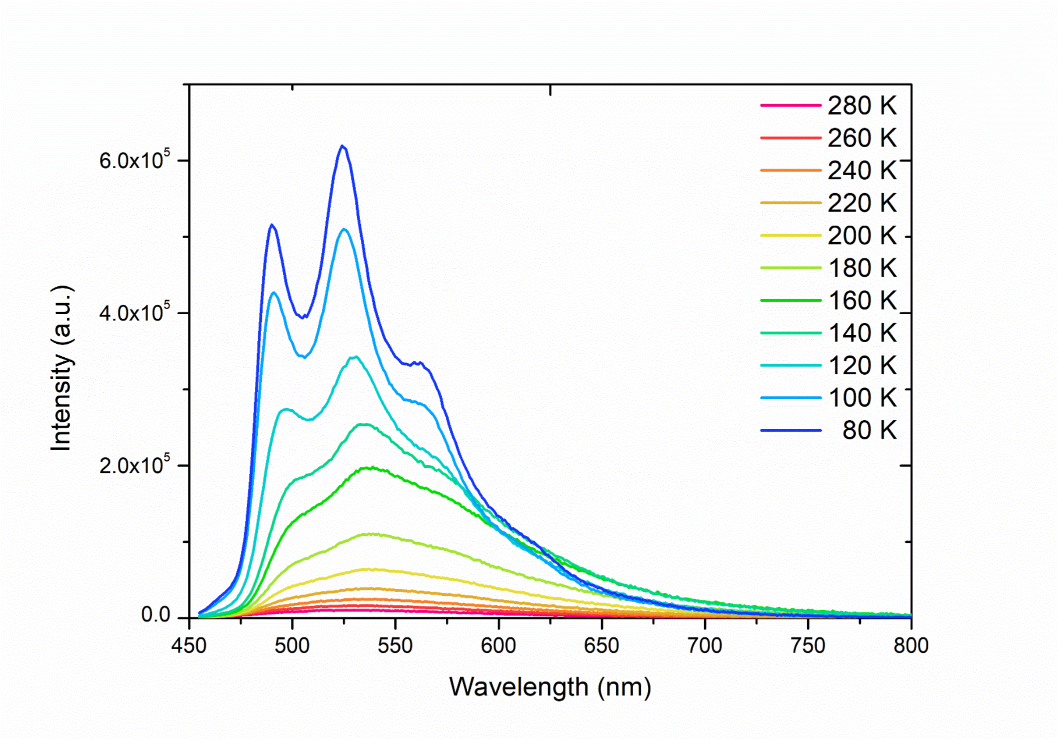

Figure S23. Temperature dependent emission spectra of stannole **4** in 2-methyltetrahydrofuran (c = 10^{-5} mol L^{-1}) during heating from 80 K to 280 K with excitation at 445 nm.

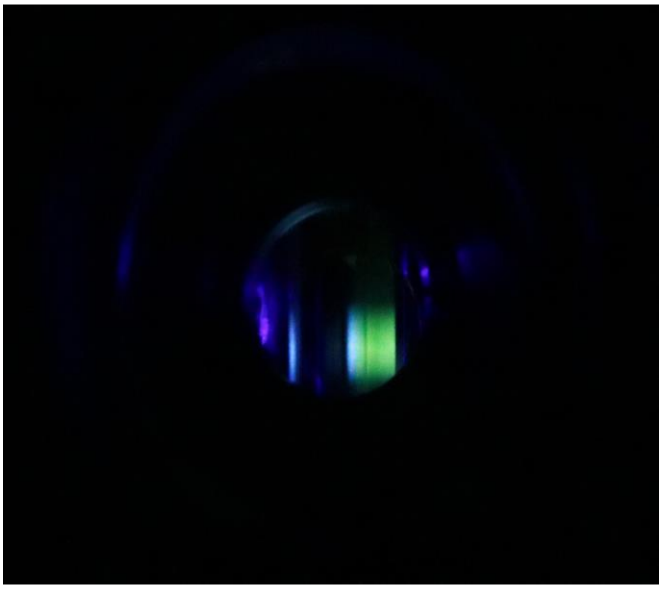

Figure S24. Irradiated sample of stannole **4** in 2-methyltetrahydrofuran (c = 10^{-5} mol L^{-1}) at 80 K with excitation at 445 nm.

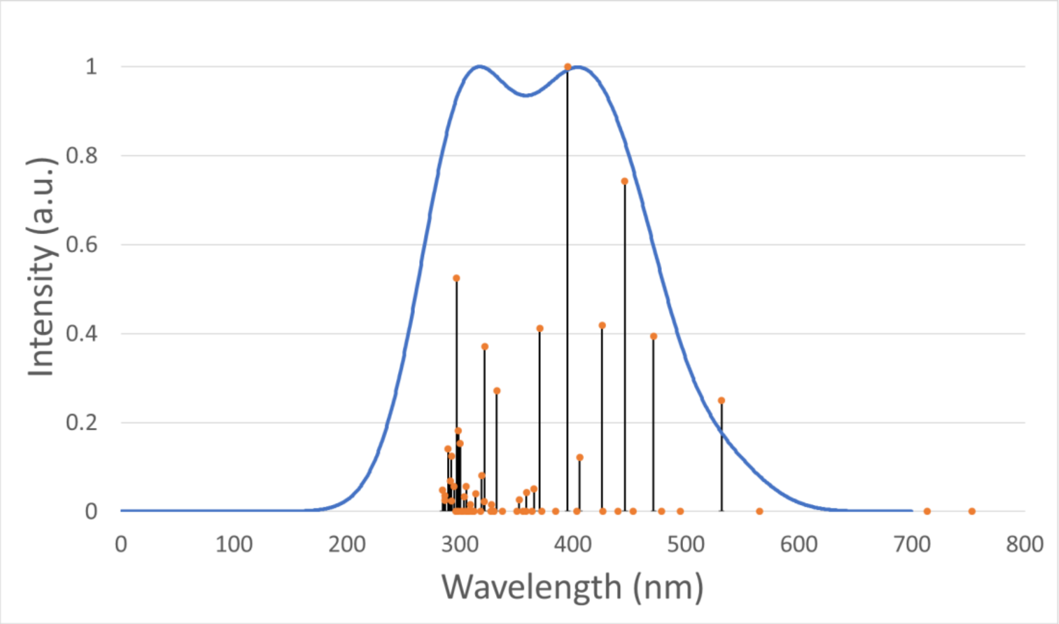

Figure S25. Theoretical absorption spectrum of lithium pentaorganostannate 5 with all transitions.

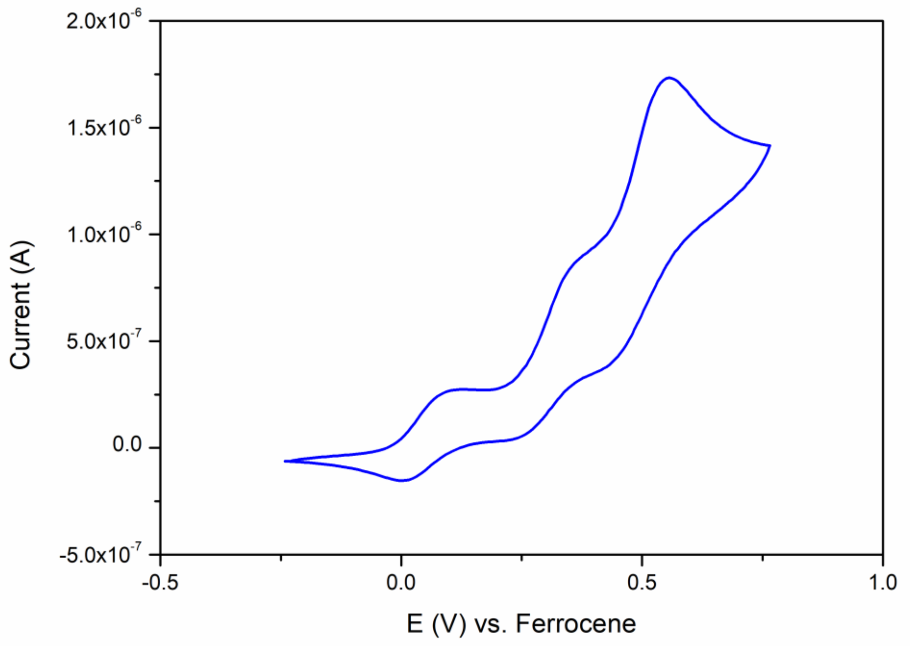

Figure S26. Cyclic voltammetry measurement of spirostannole **4** in DCM with TBA[PF_6] as conducting salt at a scan rate of 0.2 V/s.

Table S6. Significant absorption wavelengths for stannole 5 with involved transitions based on TD-DFT calculations.

Absorption wavelength	Involved transitions	CI expansion coefficients
532 nm	HOMO→LUMO<br>HOMO-2→LUMO	0.10638<br>0.68049
471 nm	HOMO→LUMO+1 HOMO→LUMO<br>HOMO-1→LUMO<br>HOMO-2→LUMO	0.18927<br>0.62113<br>-0.15123<br>0.15995
446 nm	HOMO→LUMO+1<br>HOMO-1→LUMO+1<br>HOMO-1→LUMO<br>HOMO-2→LUMO+1<br>HOMO-2→LUMO	-0.17229<br>0.11038<br>-0.12018<br>0.14520<br>0.63128
395 nm	HOMO→LUMO+2 HOMO→LUMO+1<br>HOMO-1→LUMO+1<br>HOMO-2→LUMO	-0.16771<br>0.60320<br>-0.16285<br>0.22500
297 nm	HOMO-1→LUMO+7<br>HOMO-1→LUMO+5<br>HOMO-1→LUMO+4<br>HOMO-2→LUMO+3<br>HOMO-2→LUMO+2<br>HOMO-4→LUMO+2	-0.10885<br>0.15864<br>0.54554<br>0.14677<br>-0.15580<br>0.20805

5. NMR Spectra

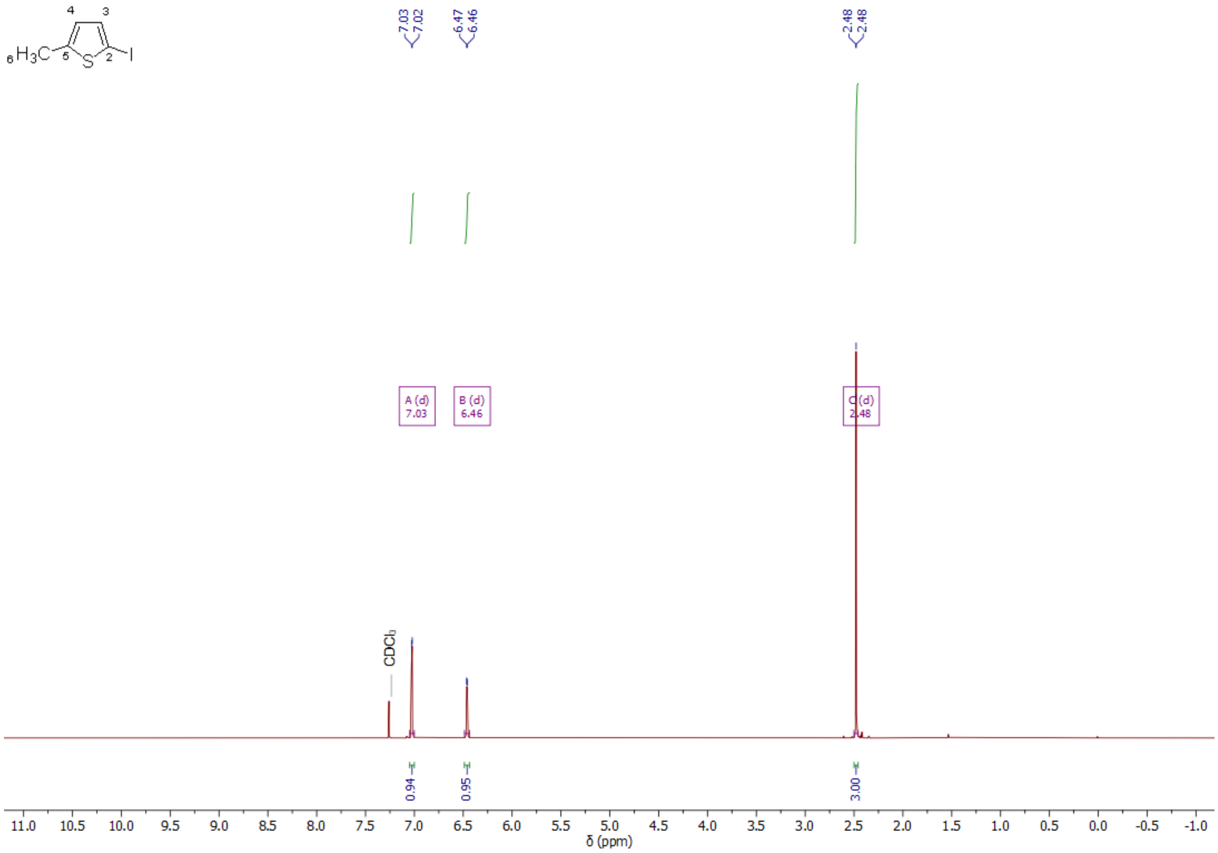

Figure S27. ^1H NMR (500 MHz) spectrum of 1 in $CDCl_3$.

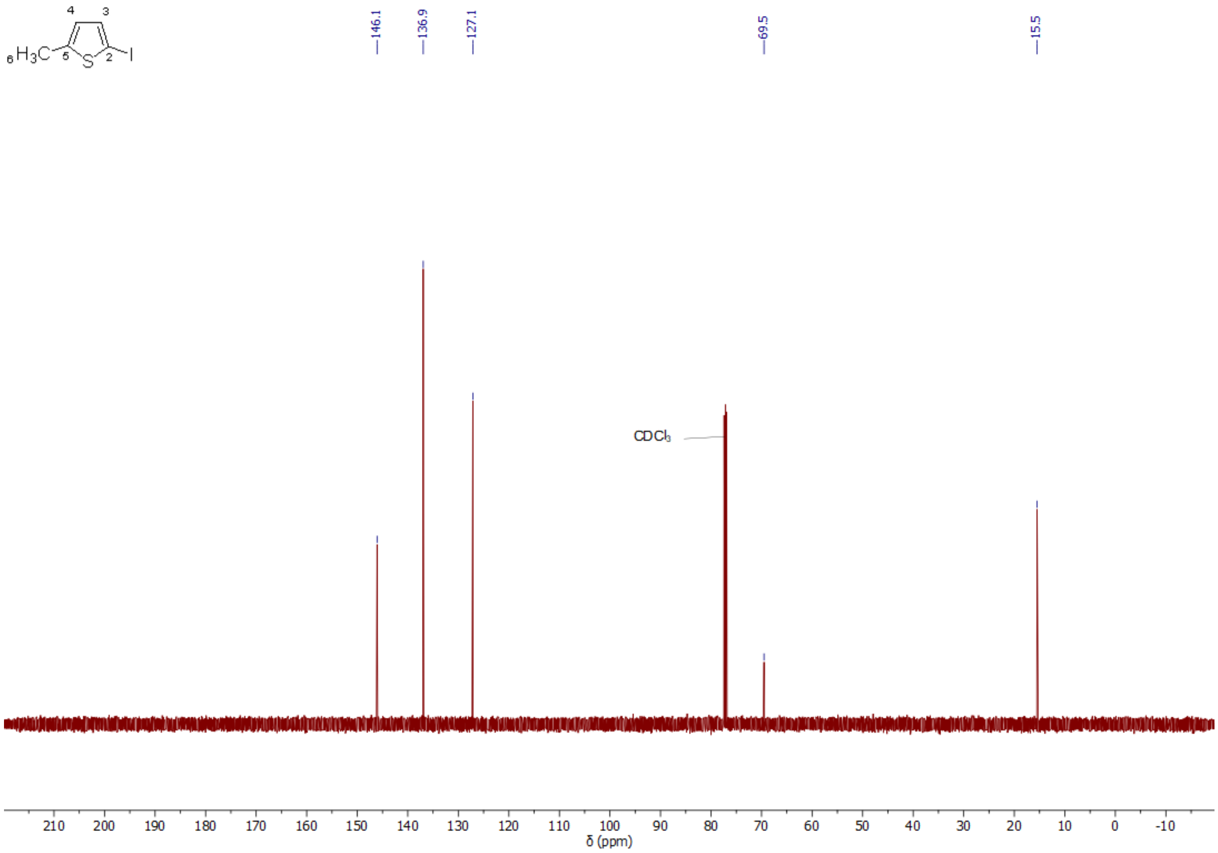

Figure S28. $^{13}C\{^1H\}$ NMR (126 MHz) spectrum of 1 in $CDCl_3$.

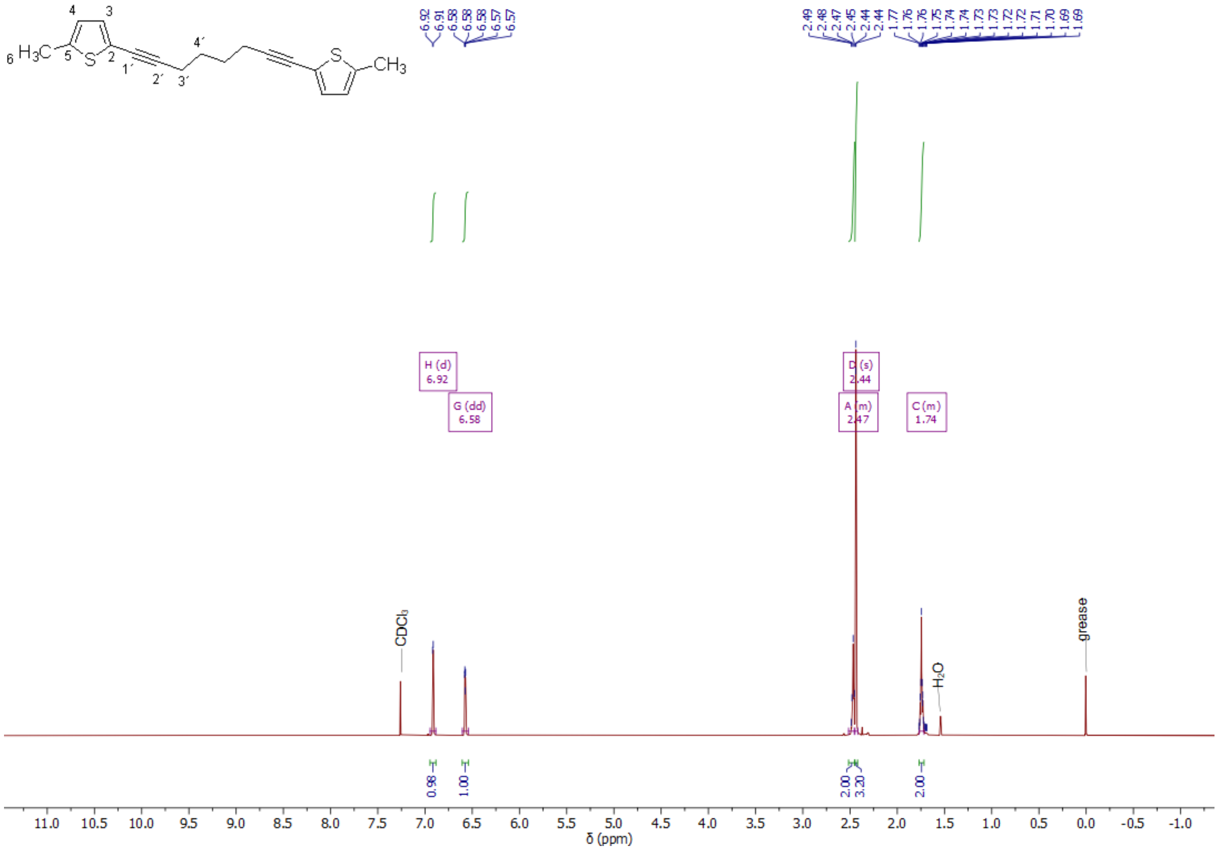

Figure S29. ^1H NMR (500 MHz) spectrum of 2 in $CDCl_3$.

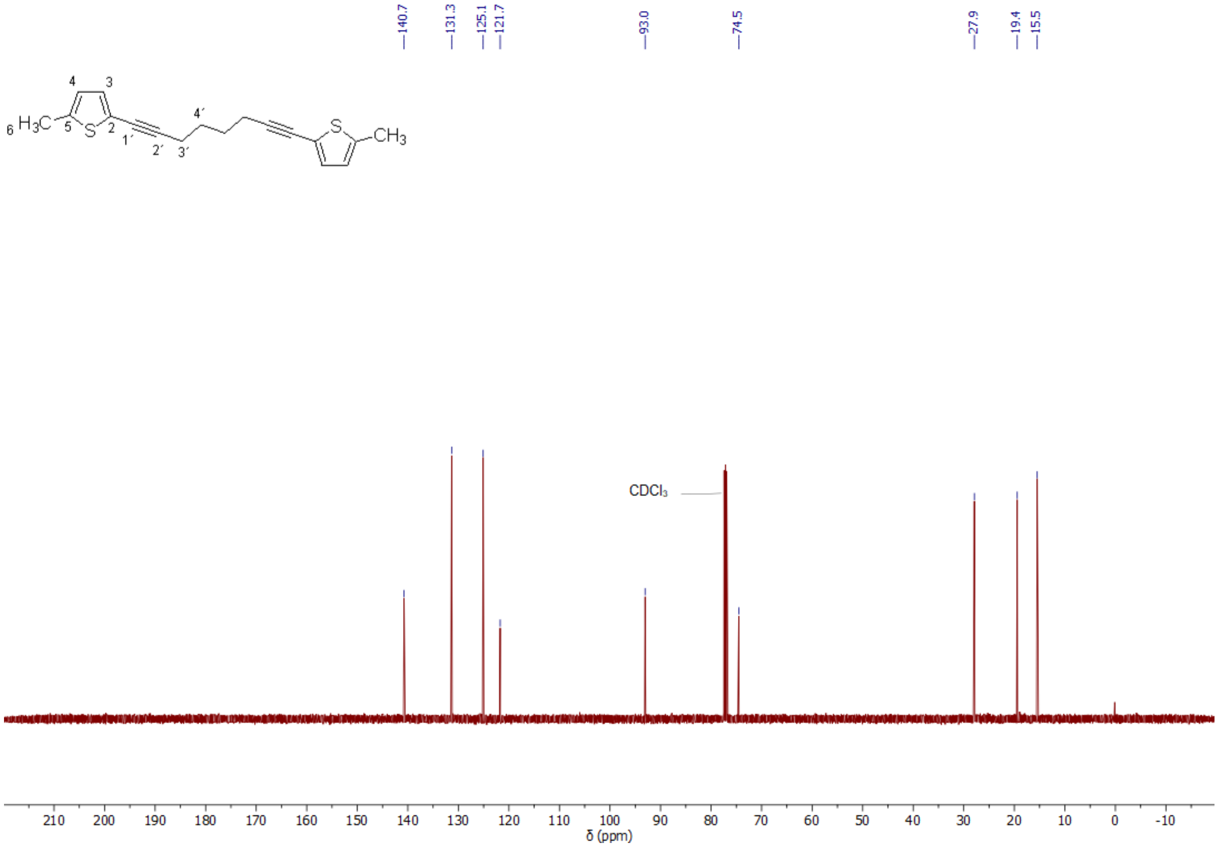

Figure S30. $^{13}C\{^{1}H\}$ NMR (126 MHz) spectrum of 2 in $CDCl_3$.

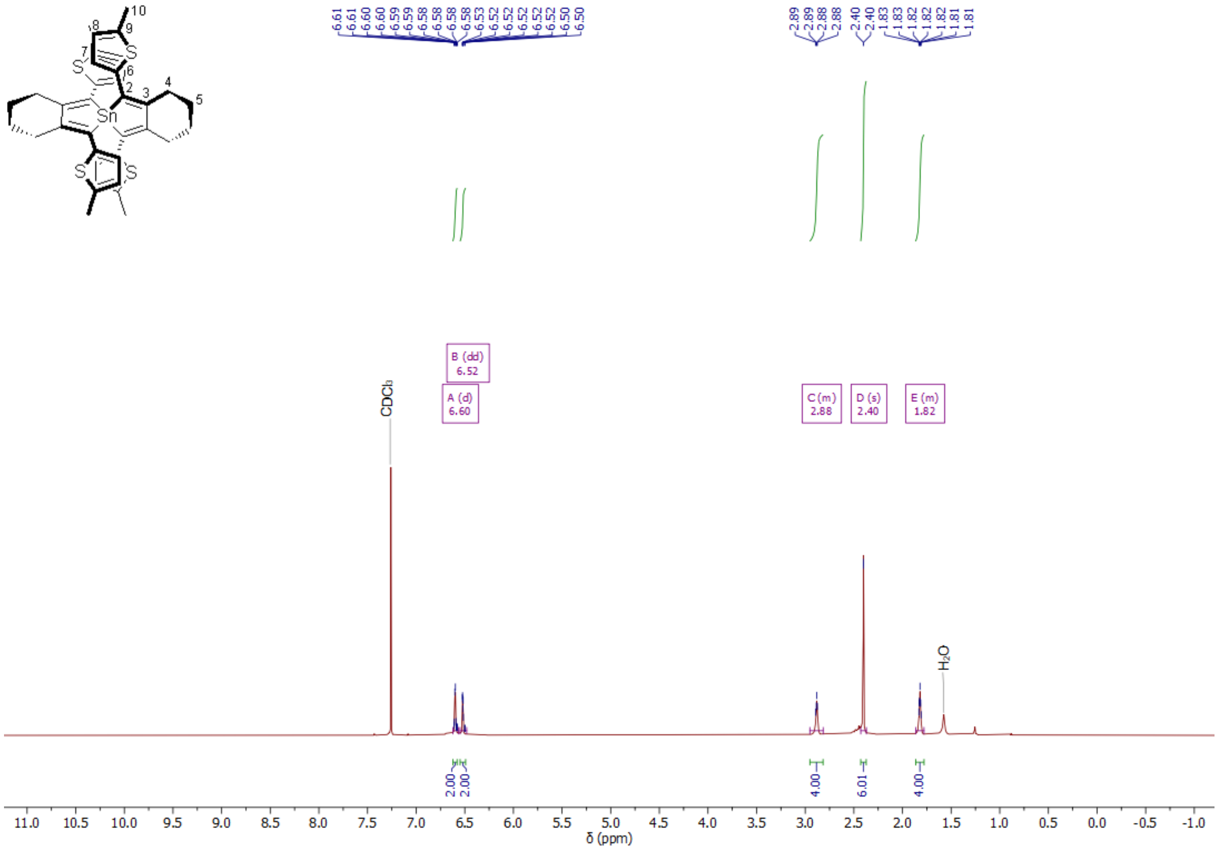

Figure S31. ^{1}H NMR (600 MHz) spectrum of 4 in $CDCl_3$.

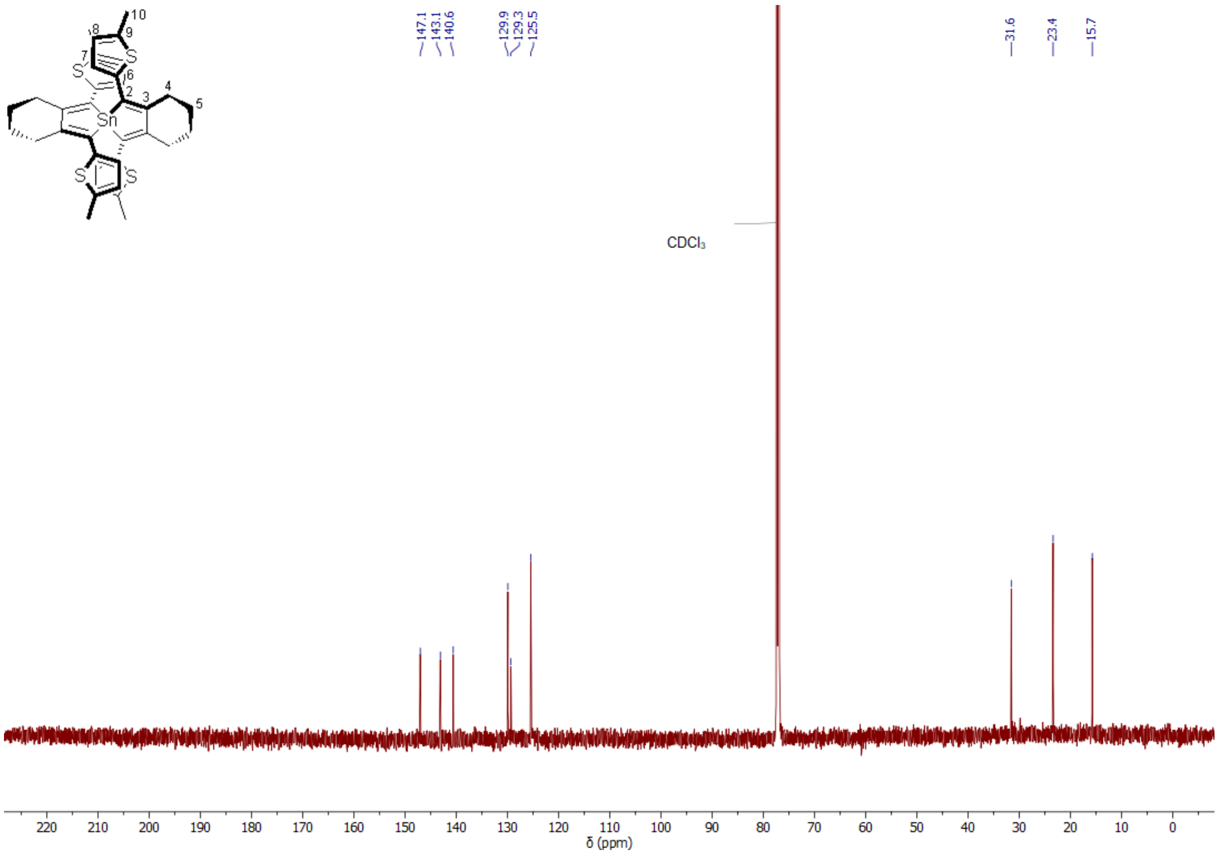

Figure S32. $^{13}C\{^{1}H\}$ NMR (151 MHz) spectrum of 4 in $CDCl_3$.

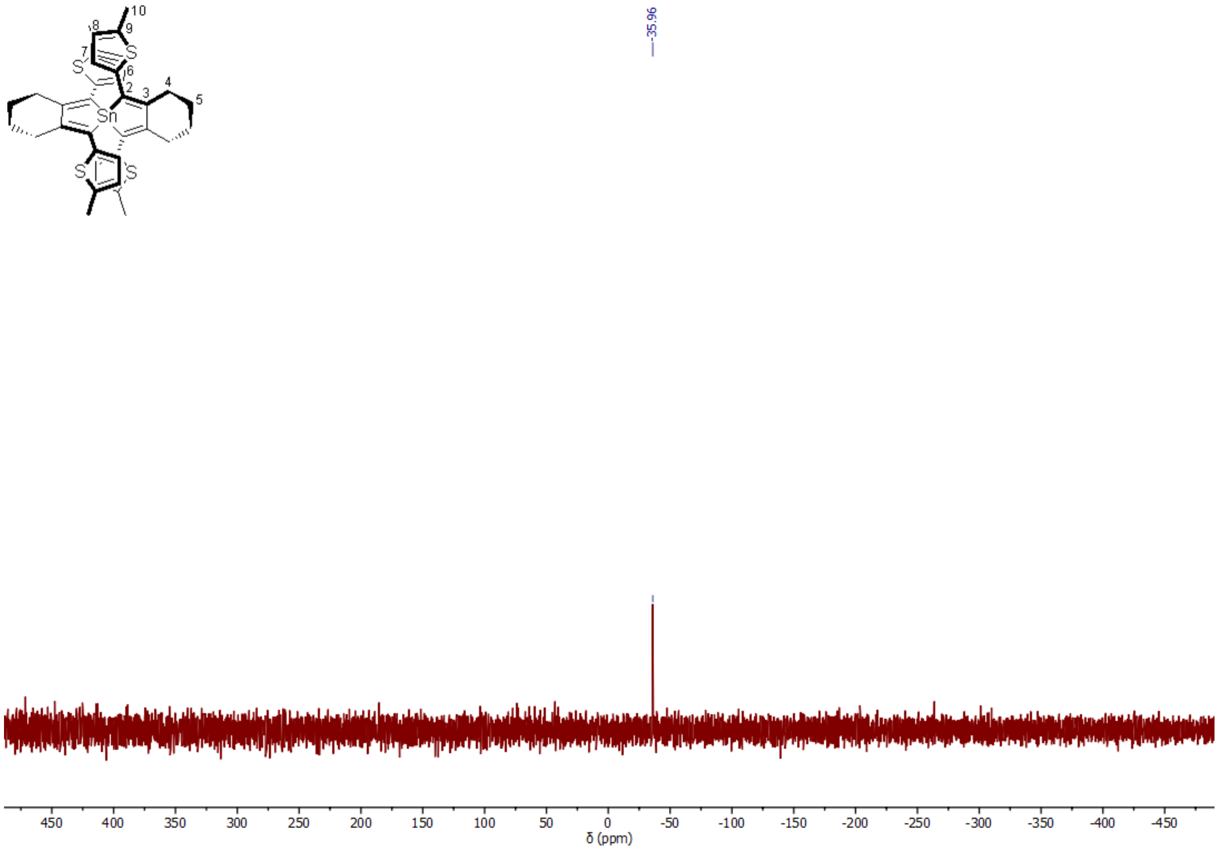

Figure S33. $^{119}Sn\{^{1}H\}$ NMR (224 MHz) spectrum of 4 in $CDCl_3$.

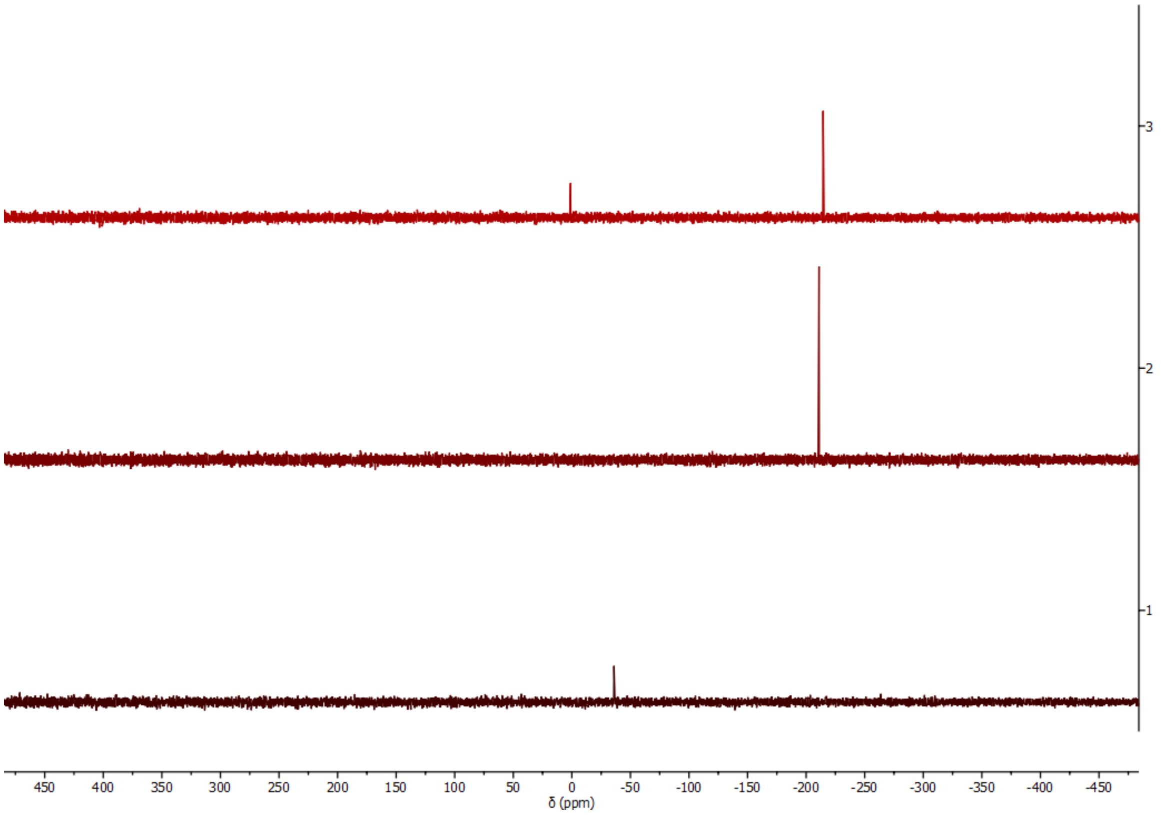

Figure S34. $^{119}Sn\{^1H\}$ NMR (224 MHz) spectrum of 4 (-36.0 ppm, bottom), 5 (-211.0 ppm, middle, -35 °C) and decomposition/ 5 (1.1 ppm and -211.0 ppm, above, 25 °C).

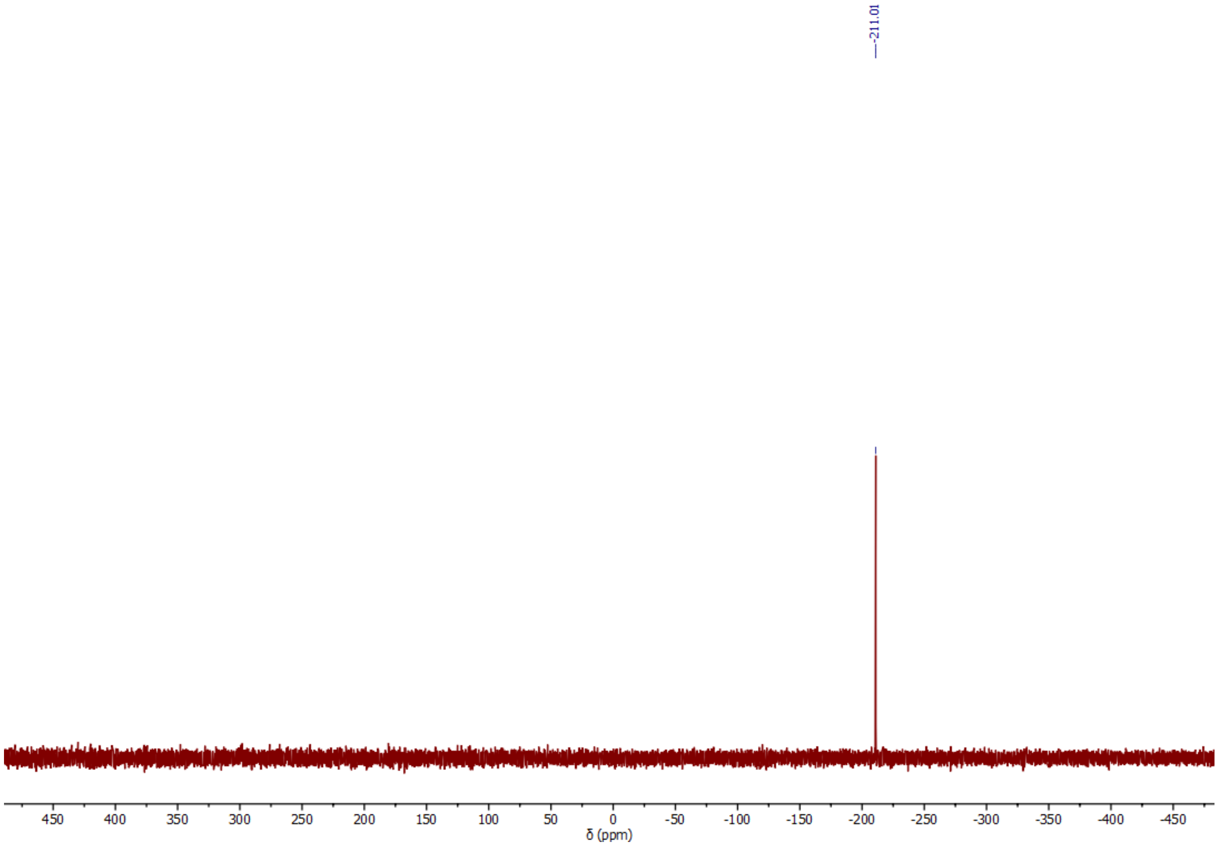

Figure S35. $^{119}Sn\{^1H\}$ NMR (224 MHz) spectrum of 5 in THF.

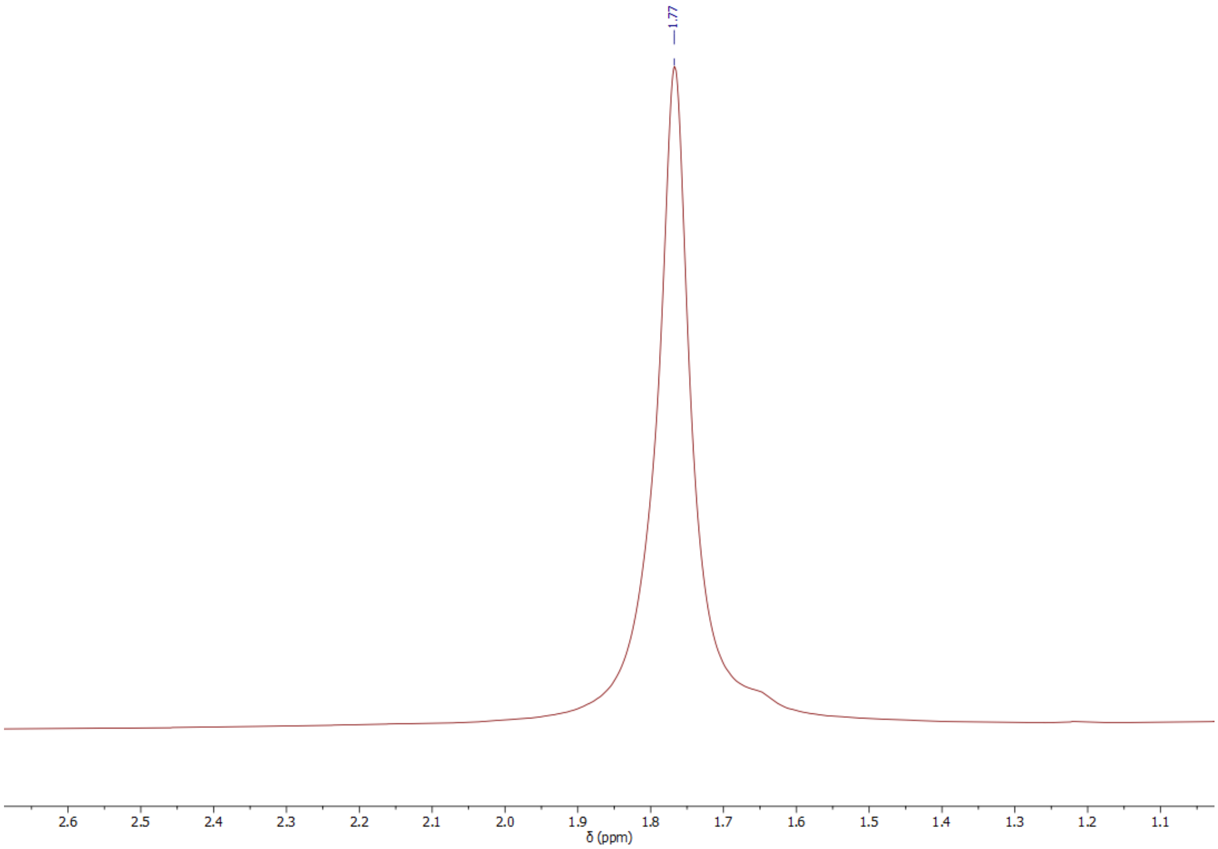

Figure S36. ^7Li NMR (233 MHz) spectrum of MeLi in THF.

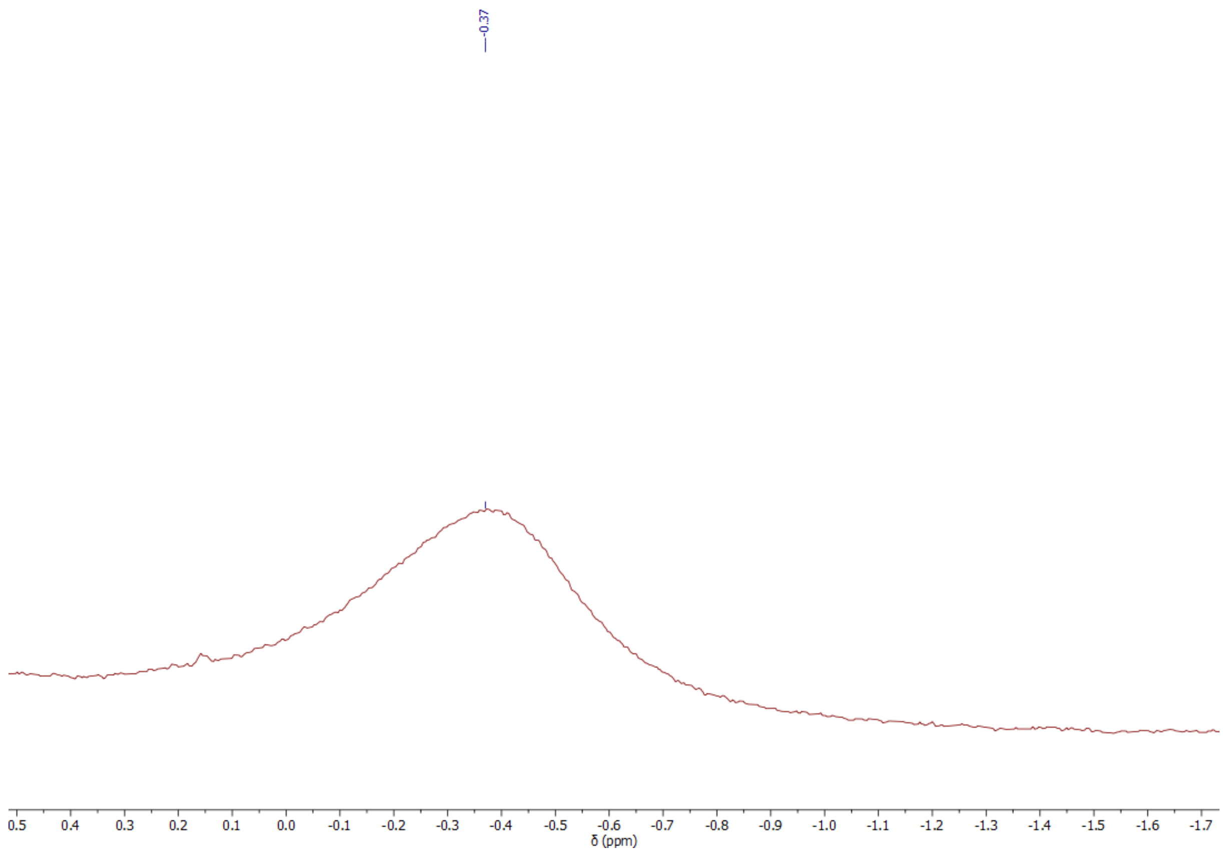

Figure S37. ^{7}Li NMR (233 MHz) spectrum of 5 in THF.

6. Literature

[1] G. Sheldrick, *Acta Crystallographica Section A* **2008**, *64*, 112-122.

[2] L. Farrugia, *J. Appl. Crystallogr.* **1999**, *32*, 837-838.

[3] O. V. Dolomanov, L. J. Bourhis, R. J. Gildea, J. A. K. Howard, H. Puschmann, *J. Appl. Crystallogr.* **2009**, *42*, 339-341.

[4] J. Grolleau, P. Frère, F. Gohier, *Synthesis* **2015**, *47*, 3901-3906.

[5] U. Rosenthal, A. Ohff, W. Baumann, A. Tillack, H. Görls, V. V. Burlakov, V. B. Shur, *Z. anorg. allg. Chem.* **1995**, *621*, 77-83.

[6] S. Urrego-Riveros, I.-M. Ramirez y Medina, D. Duvinage, E. Lork, F. D. Sönnichsen, A. Staubitz, *Chem. Eur. J.* **2019**, *25*, 13318–13328.

[7] M. Saito, S. Imaizumi, T. Tajima, K. Ishimura, S. Nagase, *J. Am. Chem. Soc.* **2007**, *129*, 10974-10975.

[8] M. Saito, S. Imaizumi, T. Tajima, *Eur. J. Inorg. Chem.* **2010**, *2010*, 2153-2157.

[9] M. J. Frisch, G. W. Trucks, H. B. Schlegel, G. E. Scuseria, M. A. Robb, J. R. Cheeseman, G. Scalmani, V. Barone, B. Mennucci, G. A. Petersson, H. Nakatsuji, M. Caricato, H. P. H. X. Li, A. F. Izmaylov, J. Bloino, G. Zheng, J. L. Sonnenberg, M. Hada, M. Ehara, K. Toyota, R. Fukuda, J. Hasegawa, M. Ishida, T. Nakajima, Y. Honda, O. Kitao, H. Nakai, T. Vreven, J. A. Montgomery Jr., J. E. Peralta, F. Ogliaro, M. Bearpark, J. J. Heyd, E. Brothers, K. N. Kudin, V. N. Staroverov, R. Kobayashi, J. Normand, K. Raghavachari, A. Rendell, J. C. Burant, S. S. Iyengar, J. Tomasi, M. Cossi, N. Rega, J. M. Millam, M. Klene, J. E. Knox, J. B. Cross, V. Bakken, C. Adamo, J. Jaramillo, R. Gomperts, R. E. Stratmann, O. Yazyev, A. J. Austin, R. Cammi, C. Pomelli, J. W. Ochterski, R. L. Martin, K. Morokuma, V. G. Zakrzewski, G. A. Voth, P. Salvador, J. J. Dannenberg, S. Dapprich, A. D. Daniels, O. Farkas, J. B. Foresman, J. V Ortiz, J. Cioslowski, D. J. Fox, *Gaussian 16 Revision A.03, Gaussian, Inc., Wallingford CT* **2016**.

[10] A. D. McLean, G. S. Chandler, *J. Chem. Phys.* **1980**, *72*, 5639–5648.

[11] R. Krishnan, J. S. Binkley, R. Seeger, J. A. Pople, *J. Chem. Phys.* **1980**, *72*, 650–654.

[12] R. C. Binning Jr., L. A. Curtiss, *J. Comput. Chem.* **1990**, *11*, 1206–1216.

[13] M. P. McGrath, L. Radom, *J. Chem. Phys.* **1991**, *94*, 511–516.

[14] L. A. Curtiss, M. P. McGrath, J. Blaudeau, N. E. Davis, R. C. Binning, L. Radom, *J. Chem. Phys.* **1995**, *103*, 6104–6113.

[15] C. Adamo, V. Barone, *J. Chem. Phys.* **1999**, *110*, 6158–6170.

[16] S. Grimme, J. Antony, S. Ehrlich, H. Krieg, *J. Chem. Phys.* **2010**, *132*, 154104.

[17] S. Grimme, S. Ehrlich, L. Goerigk, *J. Comput. Chem.* **2011**, *32*, 1456–1465.

[18] G. Igel-Mann, H. Stoll, H. Preuss, *Mol. Phys.* **1988**, *65*, 1321–1328.

[19] A. Bergner, M. Dolg, W. Küchle, H. Stoll, H. Preuß, *Mol. Phys.* **1993**, *80*, 1431–1441.
